# Synergistic Impact of Bio-Based and Waste-Derived Contents on the Mechanical and Thermal Performance of Alkali-Activated Slag Mortars

**DOI:** 10.3390/polym18161966

**Published:** 2026-08-12

**Authors:** Hakan Sarıkaya, Gülşah Susurluk, Levent Bostanci

**Affiliations:** 1 Construction Technology Department, School of Advanced Vocational Studies, Usak University, 64200 Usak, Turkey; hakan.sarikaya@usak.edu.tr; 2 Textile Technology Department, School of Advanced Vocational Studies, Istanbul Beykent University, 34520 Istanbul, Turkey; 3 Building Insulation Technologies Programme, School of Advanced Vocational Studies, Istanbul Beykent University, 34520 Istanbul, Turkey; leventbostanci@beykent.edu.tr

**Keywords:** kapok fiber, petroleum coke, waste tire rubber powder, alkali-activated slag, sustainable mortar design, waste material synergy

## Abstract

The need to enhance both the mechanical and the thermal insulation performance of alkali-activated mortars has stimulated interest in natural fiber-based reinforcement strategies, particularly for sustainability-driven design and low-carbon construction. This study explores the combined effect of natural kapok fiber, petroleum coke (PC) and waste tire rubber powder (WTRP) on the mechanical, thermal, microstructural, mineralogical, and pore structure characteristics of alkali-activated slag-based mortars. While the beneficial effects of each of these bio-based and waste-derived materials have been widely reported, little attention has been paid to their combined use and the potential for them to work together in a way that is more effective than the sum of their parts. For this purpose, 20 different mortar mixtures were produced, incorporating kapok fibers at ratios of 0%, 0.5%, 0.75% and 1%. The beneficial effect of WTRP was investigated for the case of a 3% cement additive where natural sand was substituted by PC at ratios of 0%, 10% and 20%. The results indicated that the synergistic interaction enabled the development of a sustainable mortar with enhanced thermal insulation performance of up to 37% while maintaining flexural performance compared to the control case. Microstructural, mineralogical, pore structure and thermal analyses revealed that the bio-based and waste-derived contents significantly influenced the pore network, phase evolution and fiber–matrix interactions. Overall, the results highlight the potential of the proposed sustainable material system to produce mortars with enhanced thermal insulation, sufficient mechanical performance, and lowered environmental footprint.

## 1. Introduction

### 1.1. Background

In response to increasing energy consumption, swift urbanization, and global sustainability targets, the demand for environmentally friendly materials with reduced carbon footprints and enhanced thermal insulation target in the construction materials industry is increasing daily. Cementitious materials, including concrete and mortar, rank as the second most consumed materials globally, after water, with an annual production of approximately 25 billion tons, mainly caused by their extensive application in the construction industry [1,2,3]. Global cement production is over 4 billion tons per year and is responsible for approximately 7-8% of total greenhouse gas emissions due to the high energy requirement and emissions released during the calcination of limestone [4,5,6]. This situation necessitates the development of alternative sustainable binding materials compared to conventional cement-based materials that will both reduce carbon emissions and contribute to the protection of limited natural resources [7,8].

Following the implementation of the Paris Climate Agreement, the European Union (EU) approved the “Fit for 55” package, which seeks to diminish greenhouse gas emissions by a minimum of 55% relative to 1990 levels by 2030 [9]. To achieve this target, emphasizing the reduction of carbon emissions, enhancement of energy efficiency, and implementation of circular economy strategies in energy-intensive industries, particularly in construction, has become important [10]. The incorporation of industrial waste and renewable resources in cement-based construction materials is strategically significant for reducing environmental effects and supporting the EU’s objectives for a climate-neutral economy [5]. Indeed, the combination of slag with lime in mortar originated in 1774, but the concept of alkali activation emerged in the scientific literature in the early 20th century [11]. In this context, replacing cement with industrial by-products (e.g., blast furnace slag, fly ash) through alkali activation stands out as a remarkable strategy for reducing carbon emissions and improving waste recovery [5]. Alkali-activated systems stand out with properties such as high early strength development, low permeability, good chemical resistance, and superior performance at high temperatures. Furthermore, these systems enable the utilization of industrial by-products, providing an environmentally and economically sustainable alternative. For instance, alkali-activated concrete containing fly ash and silica füme in its binder content exhibits a 69% lower environmental impact in terms of WTRP compared to conventional cement concrete [12]. In recent years, the assessment of other industrial by-products with non-pozzolanic characteristics, particularly PC and WTRP, as additives in cement-based materials has emerged as an attractive area of research, with the aim of designing building materials that are more environmentally friendly and sustainable. Although the utilization of PC in alternative binder systems has attracted interest it has been examined in only a few research studies until now [13,14]. These additives are attracting attention in the design of sustainable building materials, especially with their potential to reduce thermal conductivity, decrease density and improve certain mechanical properties such as strength-to-weight ratio and impact energy absorption capacity [15,16]. Regarding these addition types, PC and WTRP particularly emerge as significant materials in cement-based structures, including both environmental and functional aspects.

Petroleum coke is a by-product of the petroleum refining industry and contains 91–99.5% carbon in its chemical composition. Global production, which was approximately 100–150 million tons annually [17,18,19], is reported to have exceeded 150 million tons in 2019 and reached approximately 170 million tons by 2022 [19,20,21]. Its low density (approximately 0.7 g/cm^3^) and honeycomb-like, highly porous structure (up to 50%) offer significant potential for reducing thermal conductivity [22]. According to these properties, petroleum coke can be used as a fine material additive, particularly in cement-based mortars, to increase lightness and insulation performance. The modified porosity created by changing the pore structure within the mortar, together with the low thermal conductivity of petroleum coke, offers significant potential for reducing thermal conductivity [19].

Similarly, the addition of waste tire rubber particle to cement-based systems offers the potential to both recover environmentally problematic waste and improve the thermal properties of the material by reducing its density. Globally, almost 1 billion tires are discarded each year, resulting in the production of over 17 million tons of waste tires annually [16]. Incorporating these wastes, particularly in the fine fraction (≤1 mm) within cement-based materials, may lead to a 5–20% decrease in material density and an approximate 10–35% enhancement in thermal conductivity values [12,13,14]. Moreover, the porous structure and low density of waste tire rubber powders enable a reduction in the total unit volume weight of mortars to roughly 1.3–1.8 g/cm^3^, providing significant advantages, particularly in the design of lightweight insulation materials [13,14].

The incorporation of both industrial waste and renewable natural fibers in sustainable building materials is developing significance. In this regard, jute, hemp, flax and especially kapok fiber appear as materials with significant potential for reinforcement in cement-based materials due to their renewable features, biodegradability, low density, and low impact on the environment [23]. Kapok fiber features distinctive physical characteristics due to its porous and hollow structure: the void ratio is 80–90%, the density is 0.29 g/cm^3^, and the diameter ranges from 9.8 to 28 μm [23,24,25]. Due to its lumen containing a substantial volume of air, kapok fibers are highly efficient in reducing density while decreasing thermal conductivity when incorporated into cement-based materials. For instance, thermal conductivity values of 6.12 mW/(m·K) were reported in core materials consisting mainly of kapok fibers [24]. Kapok fiber is also a renewable resource. Global production is approximately 40,000–50,000 tons per year, approximately 10,000 tons of which are used in sustainable applications such as textiles and insulation. Approximately 90% of worldwide manufacturing takes place in Asia, mainly in Indonesia, the Philippines, and India. Moreover, kapok fiber, defined by its natural hydrophobic characteristics, biodegradability, and low density, becomes a significant bio-based raw material suitable for evaluation as both a structural and functional additive [25,26]. The absence of a systematic methodology in the literature about the combined utilization of materials with varying qualities, such as kapok fiber, petroleum coke, and waste tire powder, highlights an important potential area for investigating the potential synergistic impacts of these components.

### 1.2. Literature Review

The literature reveals that the addition of PC and WTRP in cementitious systems reduces the thermal conductivity values depending on the content ratio, thus significantly improving the insulation performance of the materials.

Investigations on PC additives in cement-based systems indicate positive effects, especially for thermal insulation and density reduction, while there are reported limitations on mechanical strength. Sheng et al. [27] reported that up to 20% PC addition resulted in a decrease in 28-day compressive strength of approximately 14%, while 40% addition resulted in a decrease of up to 38%. Frías et al. [28] demonstrated that the incorporation of PC enhances porosity by almost 60% and offers optimum acoustic insulation, particularly within the 2–5 mm grain range; nevertheless, this adversely affects mechanical properties. Pei et al. [29] assessed PC fly ash as a cement additive utilizing quick carbonation stabilization, achieving roughly 40% carbonation conversion and reporting a 28-day compressive strength enhancement of up to 20%. Bostancı [19] demonstrated that in mortars containing PC (0, 10, and 15% by weight), thermal conductivity decreased by approximately 33% at a 15% content ratio. In comparison to the control sample, a reduction in flexural strength of approximately 30% was found at a 10% content ratio and up to 40% at a 15% content ratio. These investigations suggest that PC may improve thermal and acoustic properties in cement and mortar systems; nevertheless, the impact on mechanical performance parameters, especially mechanical strengths, requires careful optimization.

Similarly, the incorporation of waste tire rubber powders into cement-based systems has become as a significant focus of study in recent years, targeted at both waste management and enhancement of material properties. Fadiel et al. [30] indicated that replacing 10–40% of sand with tire rubber powder resulted in a thermal conductivity decline of approximately 13–21%. Zhang et al. [14] reported substantial reductions in flexural strength resulting from diminished matrix-rubber adhesion in high-content tire rubber powder additives, highlighting that their applicability are restricted to non-load-bearing applications. Liu et al. [31] revealed gradual enhancements in thermal conductivity with 10–30% tire rubber powder additives, while also confirming the declining trend in mechanical performance as the content ratio increased. Hancharoen et al. [32] stated that there was a decrease of approximately 20–30% in flexural strength compared to the initial value at 5–15% waste tire rubber content. In general, literature findings reveal that the addition of waste tire particles (at content ratios between 5 and 40%) reduces the thermal conductivity of cement-based systems by 10–40%; however, decreases of 15–50% are observed in flexural strength due to poor adhesion at the matrix-rubber interface [30,33,34,35,36,37]. Research efforts in thermal management and advanced construction materials have increasingly focused on developing low-energy, porous, and multifunctional systems via tailored material compositions and structural designs [38,39]. Similar approaches have recently been implemented in cementitious materials to improve thermal efficiency by incorporating sustainable and waste-derived constituents. In this context, the combined utilization of hollow-structured kapok fiber, PC and WTRP represents a promising material-based strategy for enhancing the thermal insulation performance of alkali-activated mortars while simultaneously promoting resource efficiency and sustainable waste utilization.

It is observed that these content ratios mostly cause significant decreases in flexural strength, so the improvement in thermal performance cannot be sustained along with the mechanical performance. This situation reveals the need for new approaches that can improve insulation and flexural strength simultaneously. In this regard, an alternative technique to enhance the thermal and mechanical performance of cement-based systems is the incorporation of renewable natural fibers. The incorporation of renewable natural fibers into the cement matrix correlates with literature findings that significantly enhance the thermal performance of the materials, related to the fibers’ lower thermal conductivity and increased porosity [32,40,41,42,43,44,45]. However, in the present, research has examined the potential effects of increasing kapok fiber content on flexural strength and thermal insulation performance, evaluating whether a synergistic interaction between these properties exists that exceeds trends reported in the literature.

### 1.3. Research Significance

The current literature reveals that the use of PC and WTRP additions enhances thermal insulation by decreasing thermal conductivity; however, these additives generally lead to significant reductions in mechanical properties. To address this limitation, the present study developed a Li_2_CO_3_ alkali-activated slag mortar incorporating kapok fiber, PC, and WTRP to simultaneously improve thermal insulation and flexural performance. The main scientific contributions of this study are summarized as follows:

- A hybrid binder system combining Portland cement and ground granulated blast-furnace slag was employed to reduce clinker dependency.
- Petroleum coke was used as a partial replacement for natural sand, while waste tire rubber powder (WTRP) and renewable kapok fiber were incorporated as waste-derived and bio-based constituents, respectively.
- A stepwise experimental design consisting of kapok fiber, kapok fiber–PC and kapok fiber–PC–WTRP mixtures was adopted to distinguish the incremental contribution of each constituent and their combined interactions.
- The study presents a multidimensional sustainability approach by integrating clinker reduction, conservation of natural aggregates, industrial by-product utilization, waste valorization, and renewable natural fibers within a single cementitious system.

This approach significantly advances the creation of environmentally sustainable building materials with a low carbon footprint, aligning with the EU’s Fit for 55 targets, by integrating renewable natural fibers (kapok fiber) with the recycling of industrial waste (PC and WTRP). The proposed design uniquely contributes to the literature as it is the first study demonstrating enhanced flexural performance alongside reduced thermal conductivity, attributed to the synergistic interactions of industrial waste and natural fiber.

## 2. Materials and Methods

### 2.1. Materials

In this work, the hybrid binder system was designed using 42.5 R type Portland cement from Afyon Cement, Afyon, Türkiye and slag from Ereğli OYAK Cement, Zonguldak, Türkiye. Table 1 displays the chemical composition and physical parameters of the utilized cement and slag.

Standard sand of the Rilem Cembureau type, according to the TS EN 196-1 [46] standard and sourced from the Trakya Limak Cement Factory in Kırklareli, Turkey, was utilized; its density is 1.33 kg/dm^3^ and its saturated surface dry specific gravity is 2.65 g/cm^3^. Lithium carbonate (Li_2_CO_3_) provided by Sorel Kimya (Istanbul, Turkey) was used as the alkali activator.

The research also examined two distinct industrial by-products as additives: PC and WTRP. PC was sourced from Tunca Balata (İzmir, Turkey) with a particle size of 0–0.25 mm, fixed carbon content of 97%, ash content of 2%, sulfur content of 1%, and moisture content of 1%. WTRP ranging 0–0.5 mm, provided by Katek Ağaoğlu Rubber Technologies (Uşak, Turkey), was utilized. These waste products were used as additives to reduce thermal conductivity in the mortar system.

Kapok fiber, utilized as a natural fiber addition, is distinguished by its elevated void ratio, low density (0.29 g/cm^3^), and natural hydrophobic properties. The thermal conductivity of the fiber ranges from 0.04 to 0.06 W/m·K, enhancing thermal insulation in mortar systems. The characteristics of the utilized fiber are presented in Table 2. Moreover, kapok fiber offers an environmentally sustainable additive alternative due to its renewable and biodegradable properties. In this respect, the study possesses significant potential for the recuperation of industrial waste and the use of bio-based resources into construction materials.

**Figure 1:**
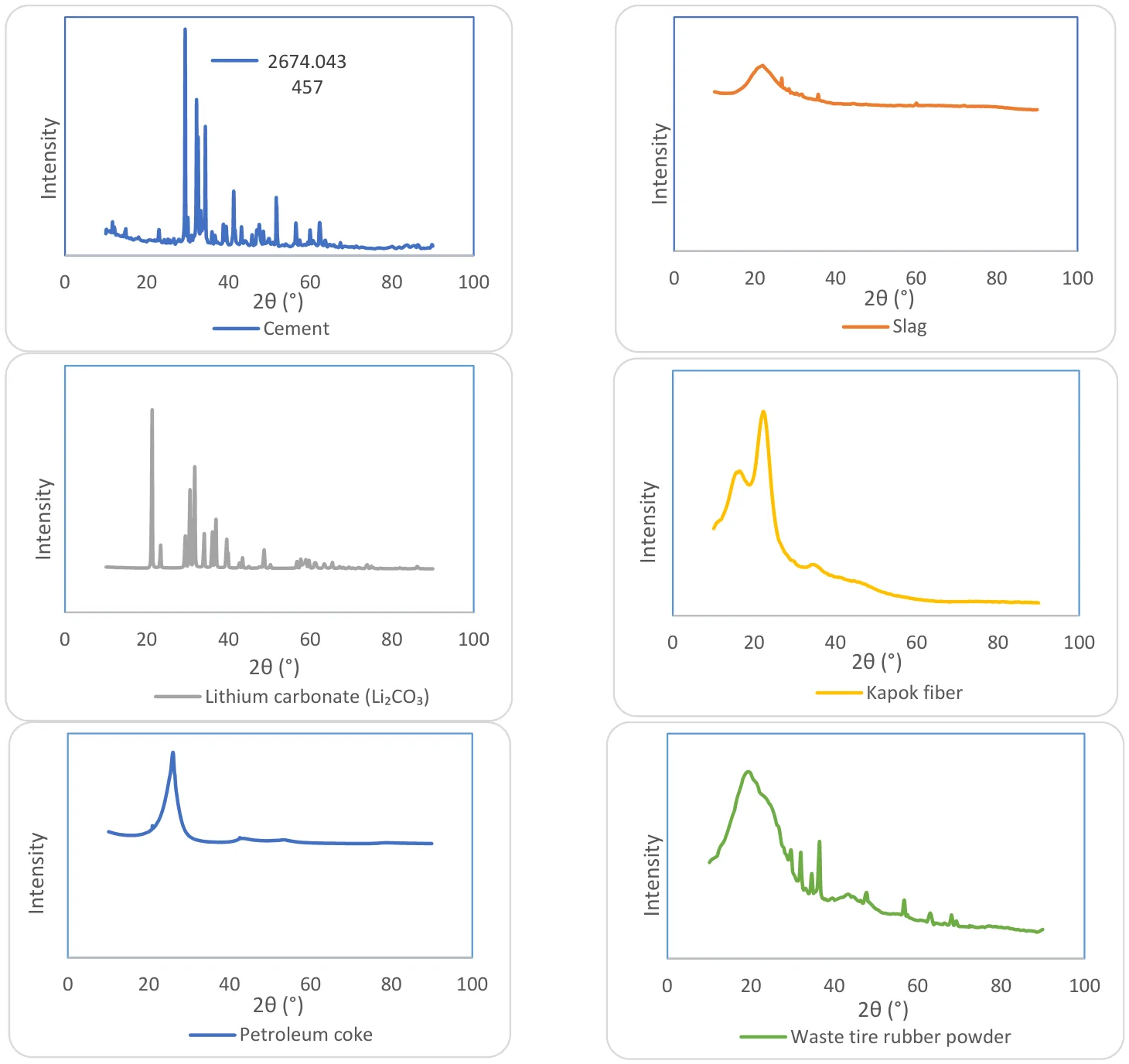
X-ray diffraction results of the raw materials.

In this context, X-ray diffraction (XRD) analysis was performed to determine the mineralogical properties of the raw materials used in the binder system. The results obtained allow for the evaluation of the reactivity potential of the raw materials and possible phase transformations within the system. The X-ray diffraction (XRD) results of the raw materials utilized in the mortar samples are illustrated in Figure 1, with the characteristic peaks identified as corresponding to the relevant phases. Tricalcium silicate (C_3_S), dicalcium silicate (C_2_S), and portlandite (CH) were observed as the main crystalline phases in the cement sample. Peaks concentrated particularly in the 2θ ≈ 29–34° range are mainly associated with the C_3_S and C_2_S phases, indicating the reactive phase content of the binder system. In the slag sample, a broad amorphous hump was detected in the range of 20–35° 2θ, rather than a definite crystalline peak, indicating that the slag is mainly amorphous and possesses a high reactivity potential. In the literature, it is known that amorphous slag dissolves during hydration and/or alkali activation processes, contributing to the formation of mainly calcium silicate hydrate (C-S-H) and aluminum-containing calcium silicate hydrate (C-(A)-S-H) gel phases. It is stated that these gel phases also significantly contribute to the improvement of mechanical and strength properties by increasing the density of the matrix [47]. The XRD pattern of kapok fiber exhibited broad, low-intensity amorphous regions rather than identifiable crystalline peaks, indicating the semi-crystalline character of fibers with a lignocellulosic structure. Similarly, the WTRP sample also exhibits low crystallinity and a broad amorphous distribution, a structure associated with the presence of elastomeric components. It is known that these types of amorphous admixtures create filler and weak interfacial effects on the microstructure rather than directly forming a crystalline phase in the cement matrix. Prominent and intense peaks were detected in the lithium carbonate sample, signifying a high level of crystallinity in the material. This crystalline structure can create possible nucleation centers that may influence the reaction kinetics within the system. The broad diffraction peak at around 25° (2θ) in the PC sample, along with the broad and low-intensity diffraction features in the 40–45° range, signifies that the material is primarily amorphous carbon and displays an irregular microstructure with a low degree of crystallinity.

These mineralogical and structural properties of the raw materials directly affect the reaction mechanisms within the binder system and the resulting hydration products. The reactivity of slag with a high amorphous phase content and the nucleation effect of additives with a high crystalline phase content play a significant role in determining the final matrix structure. The literature also emphasizes that the initial phase composition is decisive in determining the resulting gel structure and microstructure development [48]. These analyses serve as a reference for interpreting the phase formations observed in subsequent mortar samples.

### 2.2. Mixture Proportions

In this study, a total of 20 different mortar mixtures were designed and the aim of the mixtures is to improve thermal insulation performance while maintaining sufficient flexural strength. For this purpose, PC, WTRP and kapok fiber were used into the mixture designs to enhance thermal insulating qualities. The composite binder system was formulated using cement and slag, maintaining a consistent ratio of both components in all mixtures (cement: slag = 1:1). Considering the low hydraulic activity of slag, the activation of cement-slag mixtures was carried out utilizing lithium carbonate (Li_2_CO_3_). Li_2_CO_3_ was utilized at a fixed dosage of 1.5% (6.75 g), based on the findings of the authors’ previous experimental study investigating its performance as an alkali activator in slag-containing mortars [49]. The previous results demonstrated that this dosage accelerated hydration, shortened the setting time, and contributed positively to both early- and later-age compressive strength. The previous results demonstrated that this dosage accelerated hydration, shortened the setting time, and contributed positively to both early- and later-age compressive strength. Therefore, a constant Li_2_CO_3_ dosage of 1.5% was adopted in the present study to ensure consistent activation of the hybrid cement–slag binder. Accordingly, the activator dosage was kept constant in all mixtures, and the effects of varying Li_2_CO_3_ content were excluded from the scope of this study. Drinkable tap water served as the mixing material in all mixtures; the water/total binder (cement + slag) ratio was determined at 1:2, and the total binder/sand ratio was determined at 1:3.

PC was utilized as a partial replacement of sand in the design of thermally insulating cement-slag-based mortars at replacement rates of 10% and 20% by weight. The PC replacement levels were selected based on the authors’ previous experimental study, in which low replacement ratios of natural sand with petroleum coke provided a favorable balance between enhanced thermal insulation and acceptable mechanical performance. Accordingly, 10% was retained as the lower replacement level in the present study, while the upper level was extended to 20% to evaluate the performance trend at a higher but still moderate PC content [19]. Furthermore, WTRP was incorporated into the designs at a rate of 3% by weight as a cement additive. The fixed WTRP dosage of 3% was selected based on the authors’ previous experimental study conducted using a comparable cement-slag binder system. The previous findings demonstrated that a low rubber content of 3% contributed to enhanced thermal-insulation performance while limiting the associated reductions in mechanical properties. Accordingly, this dosage was adopted in the present study to utilize waste tire rubber powder at an effective but controlled level within the multicomponent mortar system [50]. The control mortar is designated by the code KP-0 as a reference. The mixtures containing kapok fiber at content ratios of 0.5%, 0.75%, and 1% (by weight of the binder) were designated as KP-0.5, KP-0.75 and KP-1, respectively. The selected fiber dosage range (0–1.0 wt.% of the binder) was determined based on the findings and experimental experience obtained from our previous studies [8,25,51]. In those studies, a maximum dosage of 1.0 wt.% was identified as providing the most favorable balance between improved thermal insulation and flexural performance while maintaining an acceptable reduction in compressive strength. PC mixes are defined as KP/10P and KP/20P, indicating substitution rates of 10% and 20%, respectively. The mixtures including PC (10% and 20%) and WTRP (3%) were designated as KP/10PR and KP/20PR, respectively. The proportions of all mixtures are presented in Table 3.

### 2.3. Sample Preparation and Experimental Methods

In compliance with the TS EN 196-1 [46] standard, three prismatic samples of 40 × 40 × 160 mm were prepared for each mixture. As is known, there are two techniques for preparing alkali-activated mixtures: the one-part and two-part techniques. The one-part technique is simpler to produce and has been more widely investigated due to its potential for large-volume construction applications. In this technique, water is added directly to a dry mixture of binder and solid activator [12]. The one-part technique was implemented when producing the alkali-activated samples for this experiment.

Kapok fibers were included after the addition of all solid ingredients to the mixture. This approach, described in the literature as the “after-mixing method,” was implemented based on the procedure suggested by Gao et al. [52]. The dry mixing procedure was conducted at a low speed, followed by the preparation of fresh mortar through the addition of tap water. The thereby produced mixes were poured into steel molds and maintained in the laboratory conditions for 24 h. After mold removal, the samples were cured in water at 21 ± 1 °C until the bending and compression tests were conducted. Flexural strength tests were conducted on samples cured for 7 and 28 days utilizing a three-point flexural test method in compliance with TS EN 1015-11 [53]. Strength values were determined by averaging three replicate measurements. The residual parts of the samples fractured during the flexural test were utilized for the analysis of compressive strength, thermal conductivity, pore structure, microstructure, and mineralogy. Microstructural examinations were performed on fragments taken from the fracture surfaces of the samples. SEM analysis was performed using a Phenom ProX desktop scanning electron microscope (SEM) located in the laboratories of the Uşak University Scientific Analysis and Technological Application and Research Center (UBATAM). Surface morphology, fiber–matrix interaction, pore structure, and distribution of hydration products of the samples were evaluated using SEM images. X-ray diffraction (XRD) analysis was performed for mineralogical characterization purposes. In this context, samples taken from those that completed their 28-day curing process were ground into powder using an agate mortar and pestle, and then sieved until a fraction below a 63 µm sieve was obtained. XRD analysis of the prepared powder samples was performed using the Rigaku MiniFlex XRD instrument located in the laboratories of the Uşak University Scientific Analysis and Technological Application and Research Center (UBATAM). CuKα radiation was used in the analysis, and measurements were taken in the 10–90° 2θ scanning range. Surface area analysis (BET) was performed to determine the pore structure. The analysis was carried out using a Micromeritics Gemini VII 2390t surface area analyzer located in the laboratories of Afyon Kocatepe University Technology Application and Research Center (TUAM), and the specific surface area, total pore volume, and pore size distributions of the samples were determined. Thermogravimetric and differential scanning calorimetry (TG-DSC) analysis was performed to evaluate the thermal behavior of the samples. The analysis was carried out using the NETZSCH STA 449 F3 Jupiter instrument located in the laboratories of the Afyon Kocatepe University Technology Application and Research Center (TUAM), and the temperature-dependent mass changes and thermal transformations of the samples were investigated.

**Figure 2:**
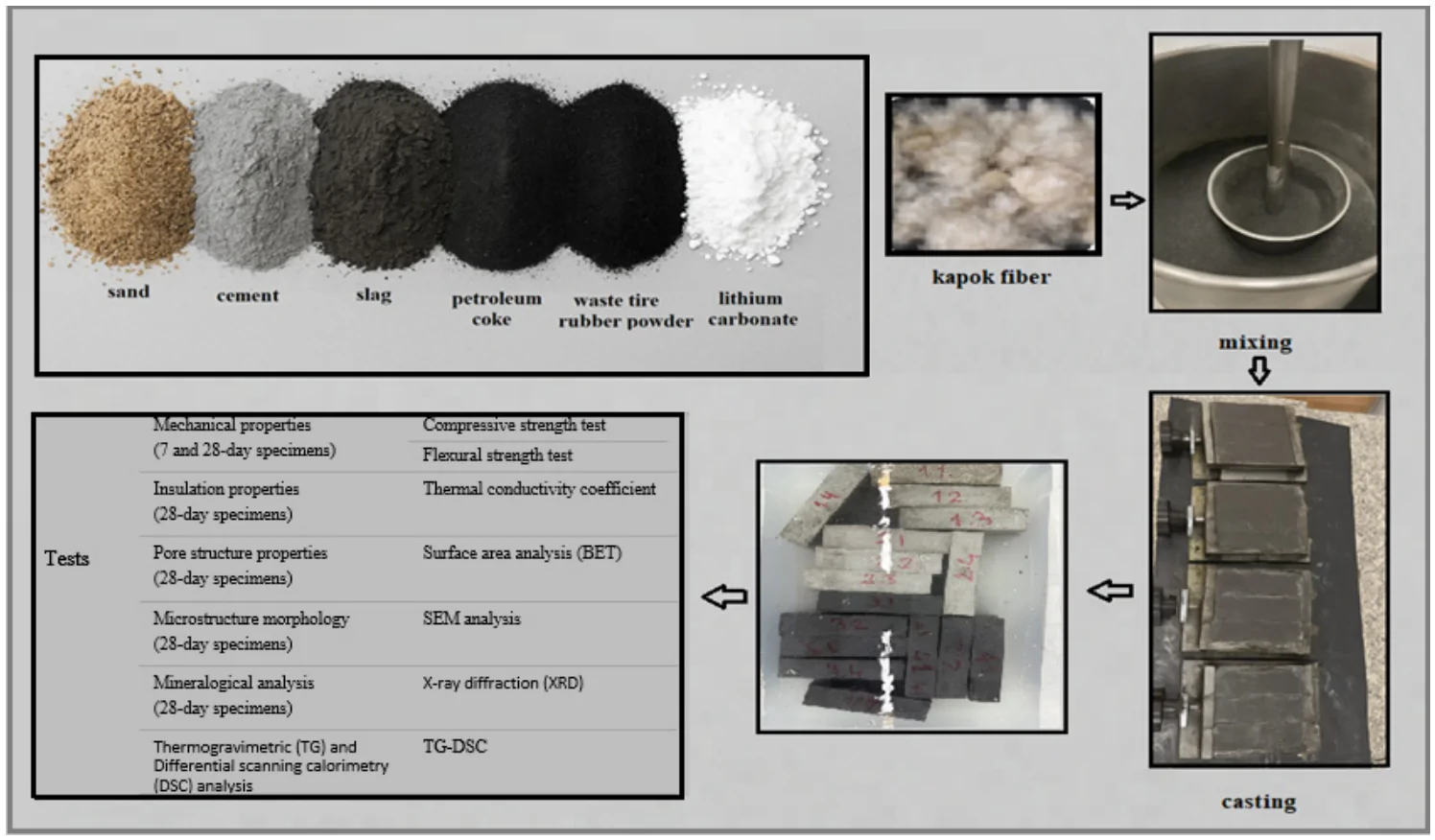
Schematic depiction of the sample preparation and testing process related to the experimental study.

Figure 2 illustrates the schematic depiction of the sample preparation and testing process related to the experimental study.

## 3. Results and Discussions

### 3.1. Compressive Strength

**Figure 3:**
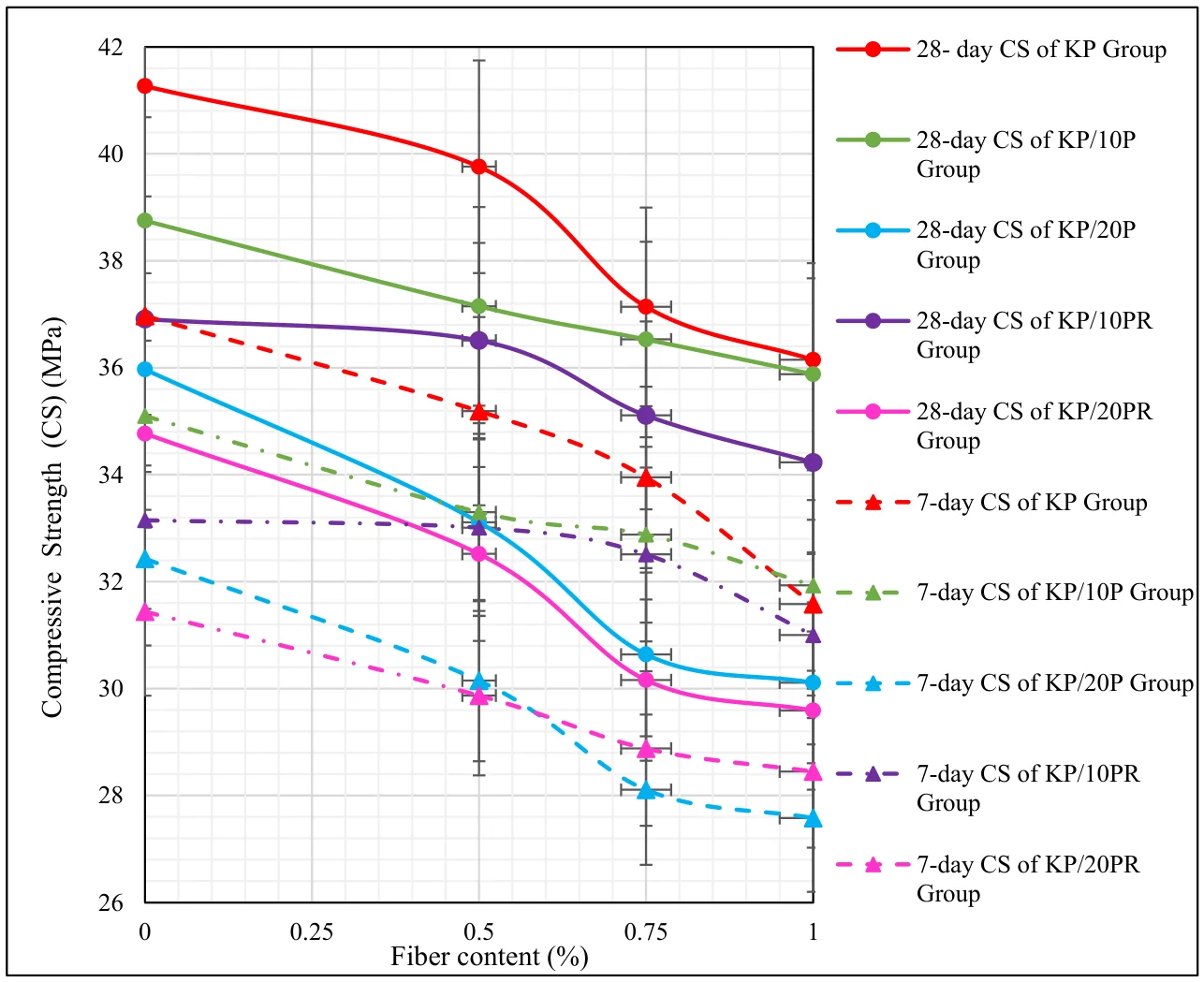
Compressive strength of mortars.

Figure 3 depicts the outcomes of compressive strength tests conducted on alkali-activated cement-slag mortars reinforced with kapok fiber, PC and WTRP after 7 and 28 days of curing.

During examination of the 7-day outcomes, it was seen that the compressive strength consistently diminished across all types of mixtures in comparison to the reference mixture. In the KP group, it was observed that as the kapok fiber content ratio increased to 0.5%, 0.75%, and 1%, the compressive strength diminished by roughly 4.81%, 8.16%, and 14.5%, respectively. Through examination of the KP/10P group, which consists of 10% PC, and the KP/20P group, which contains 20% PC, it was observed that strength losses increased with the increase in fiber content; these losses ranged from roughly 5.12% to 9.03% for KP/10P and from 7.03% to 14.95% for KP/20P. The strength losses in the KP/10PR and KP/20PR groups, which incorporated WTRP, were partially reduced in comparison to the groups containing PC. With the increase in fiber content ratio in the KP/10PR group, losses ranged from roughly 0.42% to 6.48%, whereas in the KP/20PR group, losses varied between 4.99% and 9.51%. In this context, the reduction in compressive strength is obvious in mixes containing just kapok fiber; the incorporation of PC further causes this decline. Similarly, it has been reported in the literature that PC [19,27,28] and WTRP [14,30,31,32] restrict load transfer by weakening the matrix interfacial transition zone (ITZ), hence diminishing compressive strength. However, it is understood that the combination of PC and WTRP can partially maintain matrix continuity by comparatively minimizing strength loss, particularly at higher PC content with a small amount of fiber. This effect is more pronounced for higher PC inclusion, due to the increasing tendency of porosity of the composite acquired; this means a higher volume of pores filled with fine PC particles within the interface rubber-mortar.

Following 28 days of age, it was seen that the reduction in compressive strength values became increasingly obvious compared to the control case due to the incorporation of sustainable mortar ingredients leading to higher porosity with their lower unit weight ratio characteristics. For the KP group, the increase in kapok fiber content ratio resulted in a decrease in strength of approximately 3.65%, 10% and 12.40%, respectively, compared to the reference sample. This finding suggests that the incorporation of natural fiber partially weakens matrix structure, resulting in a reduction in compressive strength as fiber content increases. The literature indicates that natural fibers limit load transfer and diminish mechanical strength by weakening the interface transition zone (ITZ) within the cement matrix [32,40,43,54,55,56]. Adhesion defects mostly occur at the fiber–matrix interface and are generally caused by the unique water absorption ability of natural fibers, which, as expected, results in reductions in compressive strength [57].

In the group containing 10% PC (KP/10P), an increase in kapok fiber content leads to a strength reduction of 4.12%, 5.72%, and 7.40%, respectively. Compared to the KP group without PC, the tendency for a reduction in compressive strength caused by fiber addition was less pronounced in samples containing PC in their matrix. On the other hand, in the group including 20% PC (KP/20P), strength reductions were more sensitive to an increase in the fiber content ratio, resulting in declines of 7.95%, 14.81%, and 16.29%, respectively. These values indicate that the combination of kapok fiber and high PC content such as 20% by weight displays the most negative impact on strength compared to all other groups examined. By examining of this trend, it is observed that the increase in PC content diminishes mortar strength significantly than kapok fiber; particularly, the 20% PC content has a critical impact. Correspondingly, the literature indicates that the incorporation of PC weakens the matrix interface surface, hence limiting load transfer and diminishing mechanical strength [20,58]. In conclusion, the presence of kapok fibers in PC-incorporated mortars may increase porosity. However, when fibers coated with PC replaced 10% of the natural sand in the mortar, they exhibited higher compressive strength and a greater tendency to reduce strength values compared to the PC-free case. Kapok fiber loading at 10% PC probably indicates better fiber interaction and bonding within the cement because adhesion defects at the fiber–matrix interface are probably lowered due to the existence of fine PC particles. This could also be an optimal content for using PC in kapok-fiber-reinforced mortar for some engineering applications, such as natural fiber-reinforced sustainable mortar.

In the group consisting of 10% PC and 3% WTRP (KP/10PR), the strength diminished by 1.08%, 4.87%, and 7.26%, respectively, as the fiber content ratio increased. These results indicate that incorporating WTRP reduces strength loss to some extent in comparison to the KP/10P group. When the KP/10PR mixture was examined, it was determined that the compressive strength decreased by 10.56% (0% fiber), 8.17% (0.5% fiber), 5.46% (0.75% fiber) and 5.31% (1% fiber) compared to the reference mixture (KP), respectively. When analyzing the difference in the contribution of rubber powder incorporated into PC, it is shown that the differences in strength decrease between KP/10PR and KP/10P are 4.46%, 1.61%, 3.82%, and 4.57%, corresponding to the fiber content ratios, respectively. This finding suggests that including WTRP at low PC levels can somewhat reduce strength loss compared to without PC, hence maintaining matrix continuity to a certain extent. The group including 20% PC and 3% WTRP (KP/20PR) exhibited strength losses of 6.47%, 13.25%, and 14.89% with increasing fiber content ratios, respectively. The investigation of the KP/20PR mixture demonstrated a reduction in compressive strength relative to the reference mixture (KP) of 15.74% (0% fiber), 18.20% (0.5% fiber), 18.79% (0.75% fiber), and 18.14% (1% fiber), respectively. While analyzing the additional reduction differences related to WTRP in comparison to PC, it is observed that the difference between KP/20PR and KP/20P is 2.89%, 1.48%, 1.29%, and 1.44% corresponding to the fiber content ratios, respectively. This finding aligns with the existing literature indicating that WTRP restricts load transmission by diminishing matrix interface bonding and can only reduce strength loss to a limited extent when combined with low unit weight additives, such as PC [14,33,36]. It is a well known fact that one of the issues with using waste rubber is that it reduces the material’s strength, which limits its use by engineers [57]. As expected, it should be noted that incorporating rubber particles into the mixture decreased the compressive strength especially at higher contents of PC. This decrease may be related to bond defects between the rubber particles and the binder matrix. Weak interfaces could act as the origin of microcracks that grow into macro-cracks, resulting in failure under compressive loading. At this point, the fact that the strength losses in the samples containing 10% PC are limited compared to the samples containing 20% PC indicates that the very fine PC particles can play a pore-filling role, similar to the KP10 group.

### 3.2. Flexural Strength

**Figure 4:**
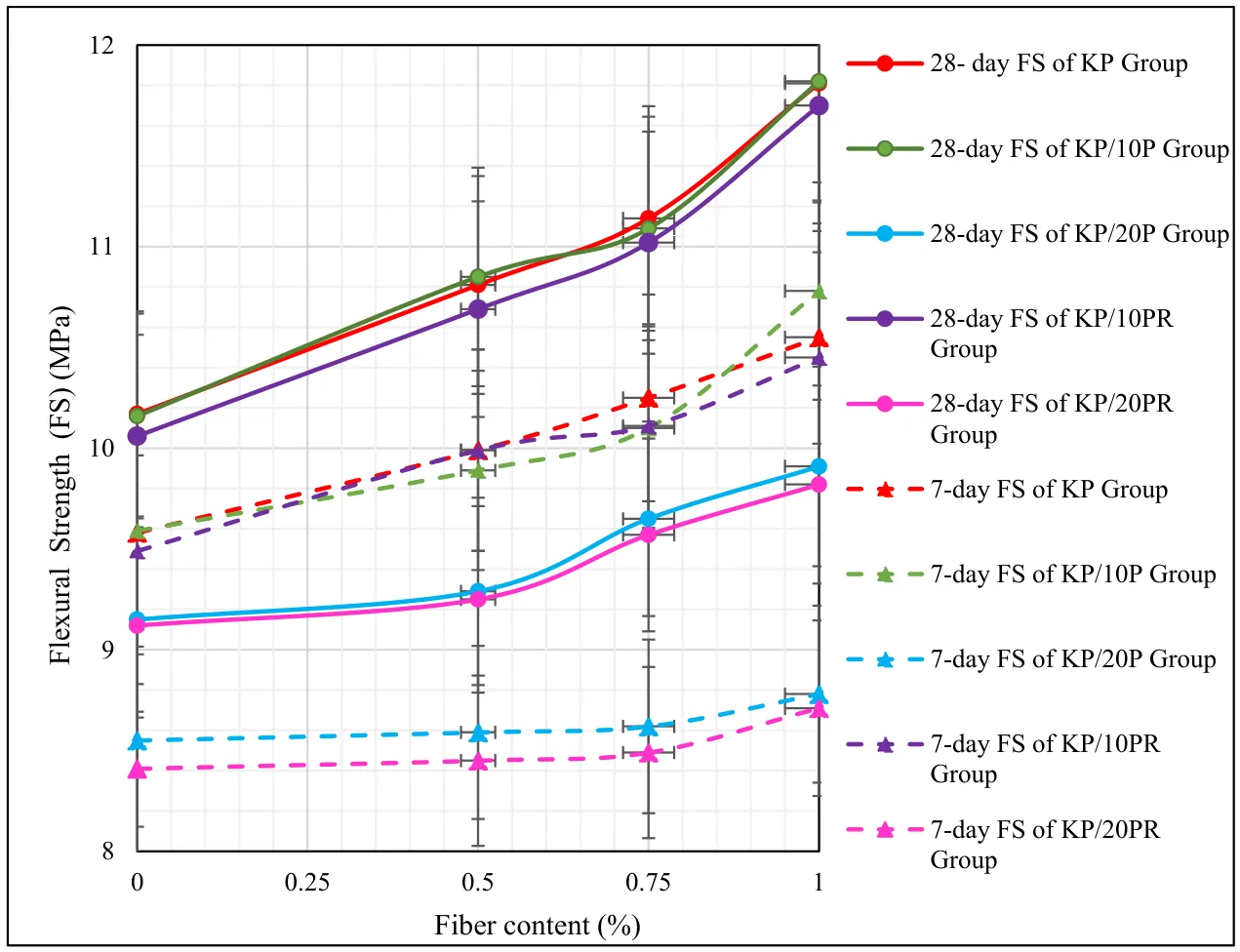
Flexural strength of mortars.

Figure 4 illustrates the results of flexural strength tests performed on alkali-activated cement-slag mortars reinforced with kapok fiber, PC and WTRP after 7 and 28 days of curing.

The addition of fibers is a common method used to improve the mechanical properties of cement-based materials, especially for enhanced flexural performance, and utilizing natural fibers due to their cost-effective and sustainability characteristics is vital [59]. When the 7-day flexural strength results were examined, it was seen that the strength increased in all mixtures containing kapok fiber compared to the reference sample. As the KP group, when the fiber content ratio increased to 0.5%, 0.75%, and 1%, the flexural strength enhanced by 4.27%, 6.99%, and 10.12%, respectively. In the evaluation of mixes including PC, the KP/10P group exhibited increases ranging from 3.12% to 12.40%, whereas the KP/20P group exhibited a more limited increase, ranging from 0.46% to 2.69%. When the mixtures containing WTRP were evaluated, the flexural strength increased between 5.26 and 10.11% as the fiber content ratio increased in the KP/10PR group, while the increases remained between 0.47 and 3.56% in the KP/20PR group. The results indicate that kapok fiber enhances flexural performance through a crack-bridging effect; however, the improvement is more restricted in mixes including high PC and WTRP. As with the compressive strength results, a continuous increase in flexural performance was observed in proportion to the fiber content. However, it is clear that the increases detected at low PC content were more significant, and that no significant increase was observed at 20% PC content, despite the addition of the same amount of fiber. As expected, the presence of a high content of PC in cement-based mixtures results in a decrease in mechanical performance in proportion to its amount due to its honeycomb-patterned porosity, non-crystalline structure, and lowest visible density [60].

The KP group exhibited a gradual enhancement in flexural strength with the increased incorporation of kapok fiber into the mixture following 28 days of curing. In comparison to the reference mixture, an increase in fiber content ratio resulted in increments of 6.29%, 9.53%, and 16.12%, respectively. In mixes of KP/10P, an increase in fiber content ratio resulted in flexural strength enhancements of 6.79%, 9.15%, and 16.33%, respectively. During the examination of mixtures containing PC, it was observed that an increase in fiber content corresponded with a significant enhancement in flexural strength within the KP/10P group (6.79–16.33%). Conversely, the KP/20P group, which had a PC content raised to 20%, exhibited a more restricted increase in flexural strength (1.53–8.30%), indicating that the high PC content partially prevented the fiber’s regeneration effect. In the combination of PC and WTRP, the fiber addition significantly enhanced the flexural strength in the KP/10PR group (6.26–16.30%), whereas the improvement was more limited in the KP/20PR group containing 20% PC (1.42–7.67%); this indicates that WTRP enhances the fiber effect at low PC contents while partially restricting the enhancement at higher contents.

Comparative evaluations of the groups reveal that kapok fiber enhances the flexural strength of the cement-slag matrix; however, industrial by-products such as PC and WTRP reduce the positive impact to an extent. Increasing the fiber ratio in the KP group consisting only of kapok fiber resulted in notable enhancements in flexural strength; this observation corresponds to the existing literature that suggests that natural fibers improve flexural performance via the crack-bridging mechanism and fiber-tensile energy [61,62,63]. It was noted that the incorporation of PC to the mixtures resulted in a more limited increase in flexural strength, despite an increase in fiber content. Particularly in the KP/20P group, which incorporates 20% PC, the enhancements achieved through fiber incorporation were limited, suggesting that PC partially affected the effective crack-bridging performance of the fibers by weakening the integrity of the matrix interface [20,58]. In the KP/10PR and KP/20PR mixtures incorporating PC and WTRP, it was found that the flexural strength enhancement provided by natural fiber was mainly maintained at a low PC content ratio (10%); however, this positive impact was significantly diminished due to the increasing weakening of matrix continuity at a higher PC ratio (20%). This is due to high waste rates generating additional voids and discontinuities within the matrix, which progressively diminishes the crack-bridging performance of the fibers. This trend aligns with the literature indicating that natural fibers enhance matrix strength via a crack-bridging mechanism that improves flexural performance [32,56], while PC and rubber-based additives introduce discontinuities and restrict load transfer by weakening the matrix interface (ITZ) bond [20,58]. The results indicate that kapok fiber significantly enhances the matrix structure and markedly improves flexural strength, even in the presence of industrial waste additives. The overall performance of various sustainable mortars in this study clearly show that replacing natural sand with 10% PC seems to be an optimum replacement level in terms of both flexural and compression performance, compared to 20% PC addition. As can be seen, considering the similar up-to-16% enhancements in flexural performance at equal fiber loadings, the matrix incorporating 10% PC acts as a PC-free matrix, given the unique but negative characteristics of PC particles with regard to strength development. This trend, interestingly, is not affected by the addition of waste tire particles, as a similar flexural performance was detected in sample KP/10PR-1.

### 3.3. Thermal Conductivity

**Figure 5:**
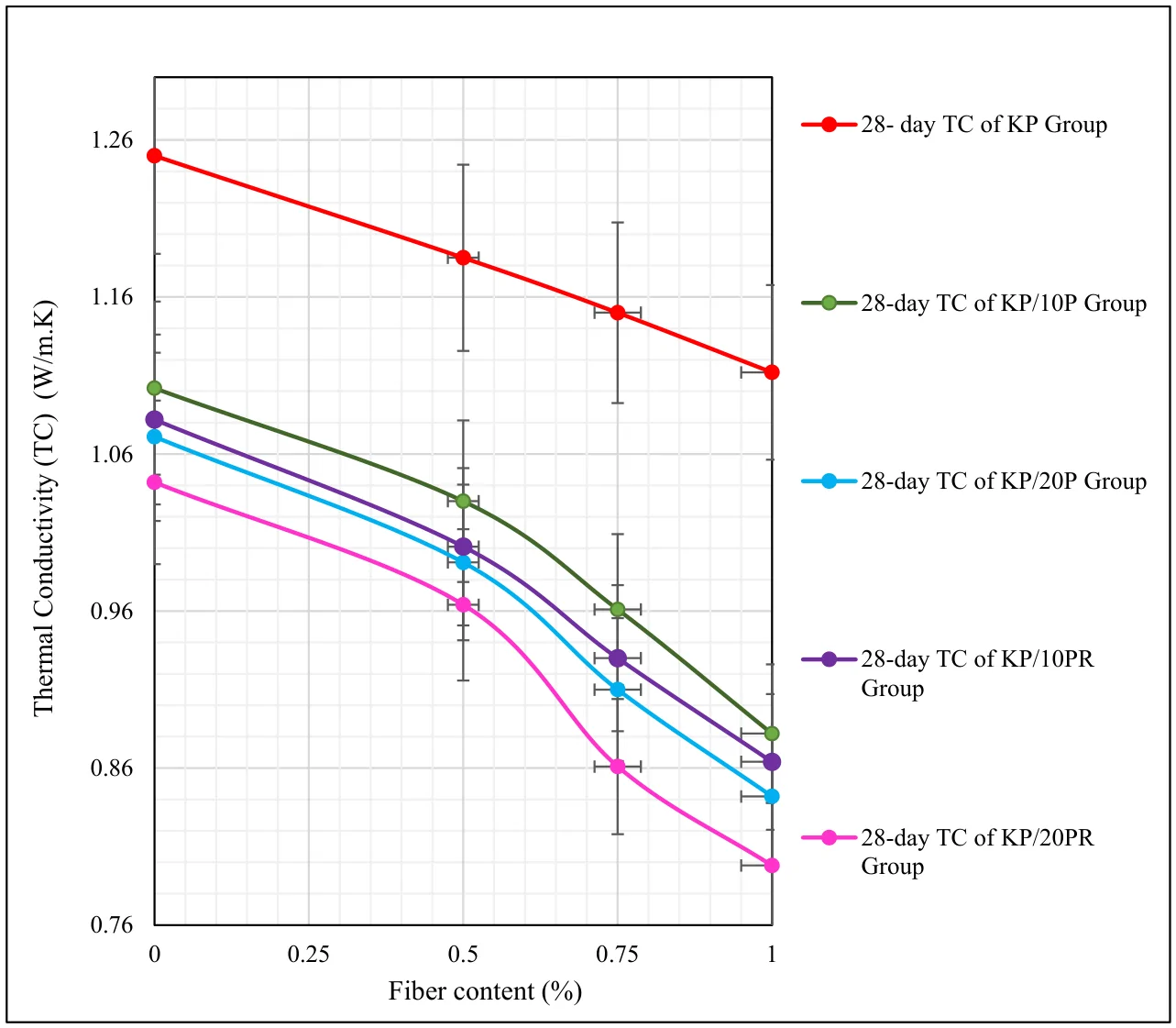
Thermal conductivity of mortars.

The outcomes of thermal conductivity coefficient assessments conducted on alkali-activated cement-slag mortars reinforced with kapok fiber, PC, and WTRP following 28 days of curing are illustrated in Figure 5.

For assessment of the KP group, a gradual decrease in thermal conductivity values was observed as the content ratio of kapok fiber in the mixture increased. In comparison to the KP-0 group, reductions of 5.20%, 8.00%, and 11.04% were observed with the inclusion of 0.5%, 0.75%, and 1% fiber content, respectively. In mixtures containing KP-10P, thermal conductivity decreased more as the fiber content ratio increased. A decrease of 6.53%, 12.79% and 19.96% was recorded at 0.5%, 0.75% and 1% fiber content ratios, respectively. In mixtures with a PC ratio enhanced to 20%, the enhancement in insulation performance became increasingly obvious with the increase in fiber content. With the increase in fiber content, there was a corresponding decrease of 7.46%, 15.03%, and 21.38%, respectively. With the increase in fiber content in the KP-10PR group, thermal conductivity diminished by 7.48%, 14.04%, and 20.14%, whereas in the KP-20PR group, it declined by 7.48%, 17.37%, and 23.41%.

When the comparisons between the groups were examined, it was seen that the addition of PC reduced the thermal conductivity of all fiber contents more than the reference mixture. Without kapok fiber (0% fiber), the incorporation of only 10% PC into the KP group diminished thermal conductivity by 11.84%; with the inclusion of 20% PC, the reduction increased to 14.32%. This trend became more pronounced with the addition of fiber; specifically, at a 1% fiber content, the thermal conductivity diminished by 24.28% in the KP/20P mixture containing only PC. When the mixtures in which PC and WTRP were used were evaluated, a decrease in thermal conductivity of 13.44%, 15.52%, 19.13% and 22.30% was observed in the KP/10PR group, and 16.64%, 18.64%, 25.13% and 28.23% in the KP/20PR group, respectively.

When the mortar sample designs within the scope of the study were examined, the mechanical and thermal conductivity of a sustainable mortar design containing half cement and half slag in the binder material was assessed, as were various multi-grade sustainable mortar designs containing different proportions of PC and WTRPs. The thermal conductivity coefficient of 1.25 W/m·K determined in the KP-0 sample enabled superior insulation performances at the levels of 12% and 14.4% in the KP/10P-0 and KP/20P-0 samples for 10% and 20% PC replacements, respectively. The addition of 1.0% kapok fiber to the KP/10P-0 and KP/20P-0 blends, which could be perceived as increasing the degree of sustainability, improved thermal insulation gains by 29.6% and 32.8% in the KP/10P-1 and KP/20P-1 samples compared to the performance detected in the KP-0 sample. The KP/20PR-1 sample, which can be considered the most sustainable mortar design within the scope of the study, indicates an insulation gain of up to 36.8% compared to the KP-0 sample, with 20% PC, 3% waste rubber, and 1.0% fiber content added to the mixtures. On the other hand, the insulation enhancements detected mostly have the following limitations: (1) The mechanism behind the insulation gain generally results in a decrease in compressive strength. (2) In samples containing equal amounts of PC or WTRP, increasing fiber content generally resulted in gains in both thermal insulation and flexural strength. However, when a 36% insulation gain was targeted, decreases in flexural strength were also observed. In this case, optimizing the mechanical strength and thermal insulation will be fundamental to the use of the mortar designs presented within the scope of the study for engineering applications.

The data indicate that the combination of PC and WTRP exhibits superior insulation performance compared to PC alone at both contents of PC, with a more pronounced impact when utilizing a high PC ratio (20%) beside WTRP. This trend aligns with literature studies indicating that PC and rubber-based additives exhibit low densities, which disrupt the continuous phases responsible for heat conduction within the matrix by introducing micro-scale discontinuities, hence diminishing overall thermal conductivity [20,30,36,58]. Similarly, it has been reported in the literature that natural fibers significantly increase the insulation performance of cement-based composites due to their low density, hollow morphology and poor thermal conductivity [40,41,43,44,45]. The integration of natural fibers with industrial waste additives facilitates the creation of innovative composite designs, providing extensive versatility and significant flexibility for sustainable construction materials.

### 3.4. Scanning Electron Microscope (SEM) Analysis

**Figure 6:**
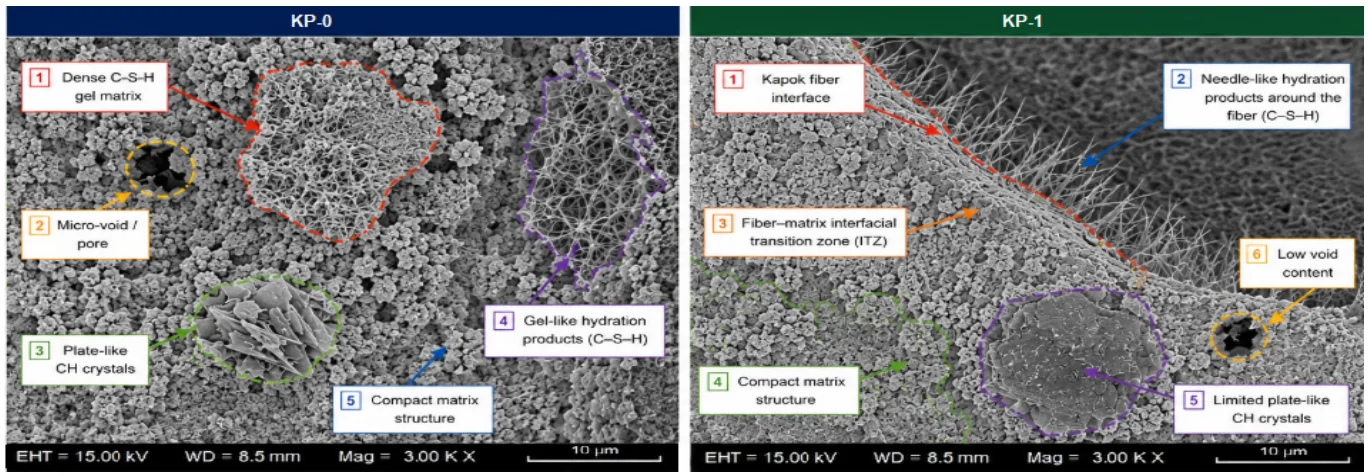
SEM micrographs illustrating the microstructural morphology of KP-0 and KP-1 mortar samples.

**Figure 7:**
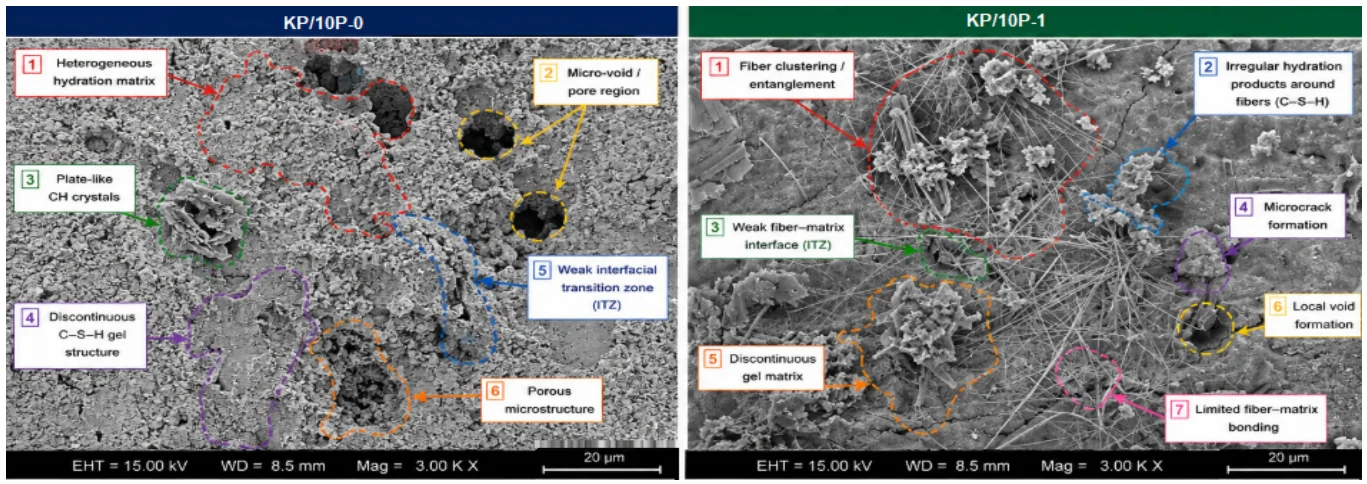
SEM micrographs illustrating the microstructural morphology of KP/10P-0 and KP/10P-1 mortar samples.

**Figure 8:**
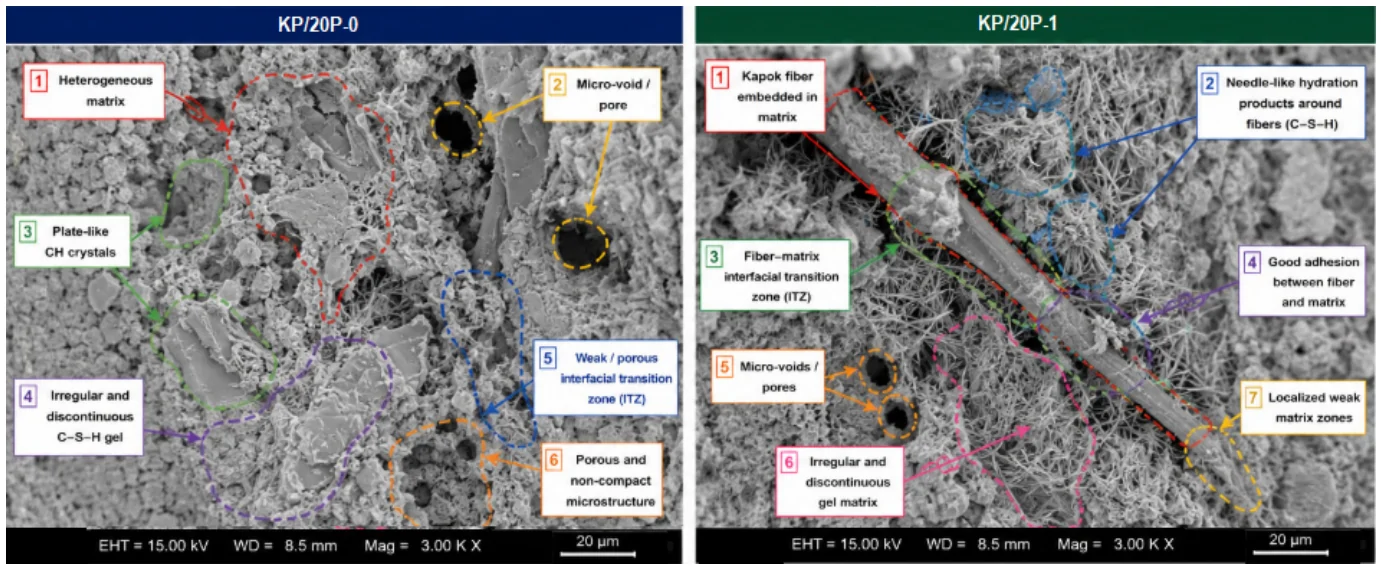
SEM micrographs illustrating the microstructural morphology of KP/20P-0 and KP/20P-1 mortar samples.

**Figure 9:**
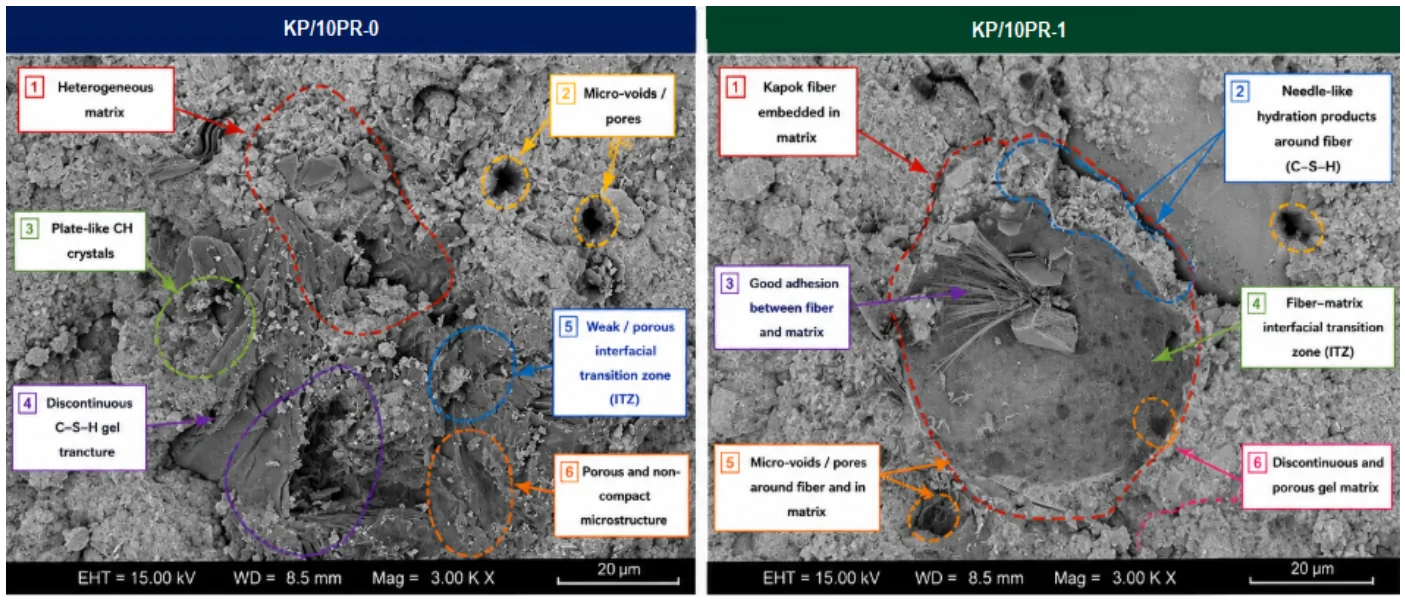
SEM micrographs illustrating the microstructural morphology of KP/10PR-0 and KP/10PR-1 mortar samples.

**Figure 10:**
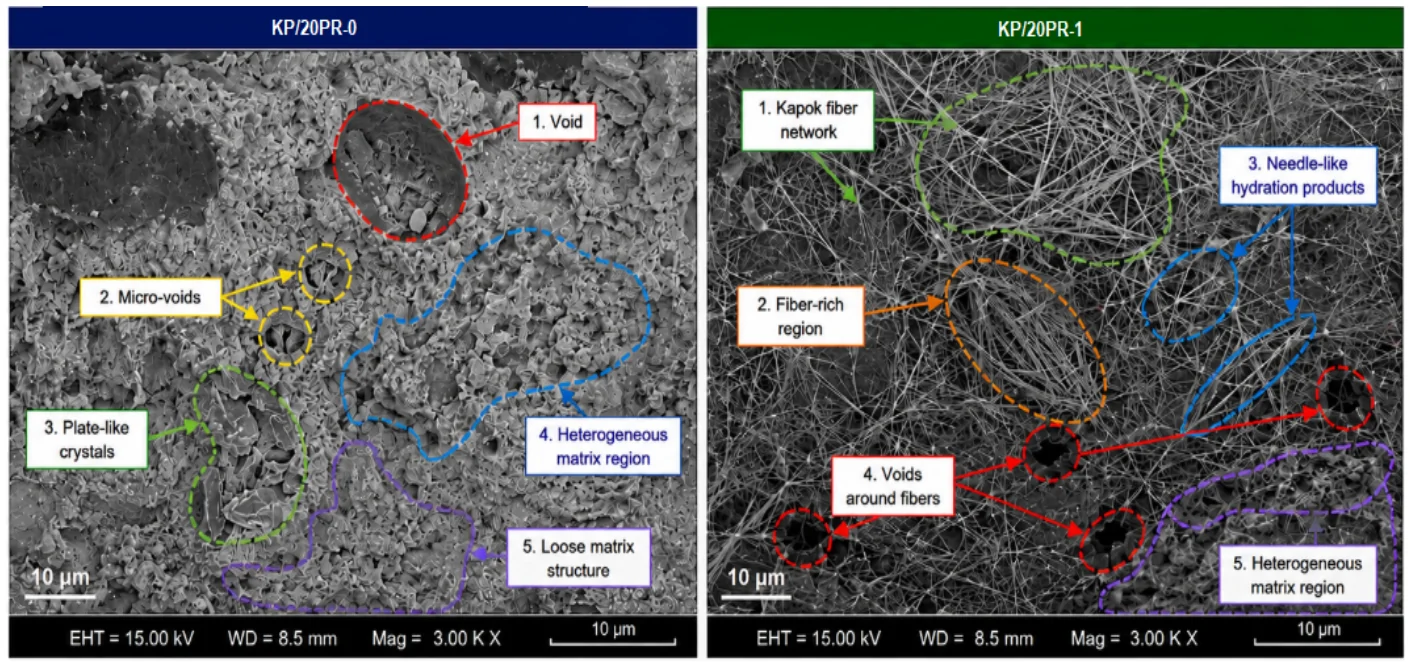
SEM micrographs illustrating the microstructural morphology of KP/20PR-0 and KP/20PR-1 mortar samples.

Figure 6, Figure 7, Figure 8, Figure 9 and Figure 10 present SEM micrographs of alkali-activated slag-based mortar samples. SEM analysis was conducted on samples without fiber addition (0% kapok) and containing a fiber addition (1% kapok), indicating the minimum and maximum thermal conductivity values in each series. SEM images illustrate the impact of PC and WTRP additives, as well as kapok fiber incorporation, on matrix structure, porosity, hydration product formation, and the fiber–matrix interface (ITZ). In the images, C-S-H gel formation, portlandite (CH) crystals, void structures, microcracks, and hydration products developing surrounding the fiber were particularly evaluated. The distribution of fibers inside the matrix and the hydration products around the fibers were evaluated in incorporated fiber samples, whereas matrix compactness, void formation and the persistence of hydration products were comparatively examined in samples without fiber incorporation.

Figure 6 displays SEM micrographs illustrating the microstructural morphology of the KP-0 and KP-1 samples. The reference sample exhibited a dense and compact matrix structure, and the gel-like formations within the matrix were consistent with the C-S-H phase determined in XRD analysis. It was observed that the kapok fibers were homogeneously distributed within the matrix and no significant voids formed at the fiber–matrix interface. The needle-like hydration products developed around the fiber indicate a strong adhesion between the fiber and the matrix. However, the limited observed plate-like crystal formations are associated with the portlandite (CH) phase identified in XRD analysis. Overall, the continuity of the microstructure, the comparatively low void ratio, and high production of C-S-H gel indicate this group generated a more stable, dense, and compact microstructure than the mortar samples.

In Figure 7’s SEM images, the microstructure of the samples containing 10% PC (KP/10P-0 and KP/10P-1) displayed a more heterogeneous, porous, and discontinuous structure in comparison to the reference sample. Microcracks, voids and irregular hydration products are commonly observed within the matrix, indicating reduced continuity of the binder gel phase and the development of a weaker microstructure. The plate-like crystal formations that are prominent in places in the images are associated with the portlandite (CH) phase, and the relative increase in these crystals indicates that the compact C-S-H gel structure has not developed sufficiently. The literature also indicates that a reduction in the gel phase and an increase in CH crystals correlate with microstructural looseness, increased porosity, and decreased strength [47].

The inert characteristics of PC particles is thought to result in limited bonding with the matrix, causing voids to develop around the particles and creating weaknesses in the interface transition zone (ITZ). Similarly, it has been reported in the literature that low-reactive additives disrupt matrix continuity, increasing porosity and negatively affecting mechanical performance [64].

In regions with incorporated fibers, it was seen that the fibers exhibited a tendency to aggregate in some areas inside the matrix, micro-voids developed around the fibers, and hydration products accumulate irregularly on the fiber surface. This indicates that the fiber–matrix adhesion is weaker compared to the reference sample, and the crack-bridging effect of the fibers may be limited. This group of samples exhibited a lower compact microstructure characterized by an increased void ratio, prominent CH crystals, weak particle/fiber–matrix interaction, and discontinuous gel structure, which is consistent with decreased binder system effectiveness and loss of mechanical performance.

Figure 8 displays SEM micrographs showing the microstructural morphology of samples containing 20% PC (KP/20P-0 and KP/20P-1). It was observed that the microstructure of these samples displayed more heterogeneity and occasionally discontinuity relative to the reference group. Samples without fiber inclusion, plate-like crystal formations, and void regions are noteworthy, and these structures are considered to be associated with the portlandite (CH) phase. Furthermore, irregular gel formations and discontinuities were observed within the matrix, indicating that the binder gel phase did not develop homogeneously. In the fiber-containing sample, it was determined that kapok fibers were positioned within the matrix and that needle-like hydration products developed densely around the fibers. These structures are thought to indicate increased adhesion at the fiber–matrix interface (ITZ) and the formation of nucleation sites for the hydration products around the fibers. The literature also indicates that natural fibers promote the development of local hydration products due to their surface properties [65]. However, micro-voids, discontinuities, and areas of weak matrix were also observed in the images. This is considered to be related to the fact that the increasing content of PC, due to its inert character, limits hydration reactions and weakens matrix integrity. The literature also indicates that the use of inert additives in high content enhances porosity, resulting in weakened microstructure [64].

In conclusion, while the incorporation of fiber led to localized microstructural enhancements and improved adhesion around the fibers, the presence of voids and matrix discontinuities due to the elevated content of inert reinforcement prevented the development of a fully compact microstructure.

Figure 9 displays SEM micrographs showing the microstructural morphology of samples (KP/10PR-0 and KP/10PR-1) containing 10% PC and 3% WTRP. SEM images revealed that the microstructure of these samples exhibited a distinctly heterogeneous, porous and discontinuous character. Samples without fiber incorporation exhibit wide void regions, discontinuous gel structures, and plate-like crystal formations. These crystal structures are considered to be related to the portlandite (CH) phase identified in XRD analysis. Moreover, it is observed that a compact and continuous C-S-H gel structure has not been fully developed within the matrix. In the fiber-containing sample, it was observed that kapok fibers were effectively positioned within the matrix and that dense needle-like hydration products developed on the fiber surface. These formations indicate that the fibers create nucleation sites for hydration products and increase adhesion at the fiber–matrix interface. Furthermore, the fact that some fibers extend along the gap/crack regions reveals that the fibers contribute to the microcrack-bridging mechanism. However, the porous and discontinuous structure observed throughout the matrix suggests that increasing inert additive content limits the continuity of the binder gel. It is believed that due to the inert/hydrophobic character of WTRP and PC, they cannot form effective bonds with the matrix, resulting in the formation of voids around the particles, weak interface zones (ITZs), and microstructural discontinuities. Literature reports also indicate that in tire-modified systems, hydration products do not develop sufficiently on the tire surface, leading to high porosity and poor bonding [13]. Furthermore, increasing the content of inert additives leads to a decrease in C-S-H gel formation and matrix compactness, resulting in losses in mechanical properties [15].

In summary, the incorporation of fiber in these samples enhanced local adhesion and the formation of hydration products surrounding the fibers; however, the simultaneous use of PC and WTRP additions diminished the continuity of the binder gel phase, leading to a more porous and less dense microstructure. This finding, when considered together with the SEM and XRD results, is consistent with a decrease in mechanical strength.

Figure 10 depicts SEM micrographs illustrating the microstructural morphology of samples (KP/20PR-0 and KP/20PR-1 including 20% PC and 3% WTRP). SEM images revealed that the microstructure of these samples exhibited a heterogeneous and porous character. In the samples without fiber incorporation, wide void regions, discontinuous gel structures and occasional plate-like crystal formations are noticeable within the matrix. These structures are considered to be consistent with the portlandite (CH) phase identified in XRD analysis. Furthermore, it is observed that the binder matrix fails to form a compact and continuous structure. In the fiber-containing sample, it was observed that kapok fibers were densely distributed within the matrix and fine needle-like hydration products developed along the fiber surfaces. This indicates that the fibers generate nucleation regions for local hydration products. However, the presence of porous regions and weak interfacial zones (ITZs) around the fiber indicates that the fiber–matrix bond is not sufficiently developed in all regions.

The dispersed and discontinuous hydration products observed in the images indicate that the binder gel phase developed homogeneously. This situation is thought to be related to the limitation of hydration reactions due to the inert/hydrophobic character of PC and WTRP. Literature reports also indicate that PC additives increase porosity and reduce matrix compactness [58], and that tire rubber powder creates weak interfacial regions, leading to microstructural discontinuities [60].

In short, although fiber reinforcement facilitated the formation of localized hydration products in these samples, the high inert additive content weakened the continuity of the binder matrix, leading to a more porous microstructure. This finding aligned with the XRD measurements and the observed reductions in mechanical strength.

Microstructure analysis reveal a significant transition from the dense and well-bound matrix structure observed in the reference sample to a progressively more heterogeneous and porous structure with the increasing addition of PC and WTRP additives. In the reference group, a compact microstructure characterized by a predominant C-S-H gel structure and strong interfacial bonding was observed, indicating efficient hydration and high structural integrity. The incorporation of PC led to a disruption in matrix continuity owing to the inert character of the particles, resulting in an elevated void ratio and the formation of weak interfacial transition zones (ITZs). While local enhancements were noted with elevated PC content and fiber reinforcing via nucleation and crack-bridging mechanisms, the increased PC content constrained this beneficial effect. The combined use of PC and WTRP further weakened the microstructure; the hydrophobic and inert nature of the tire rubber powder further reduced interfacial bonding, while the increased inert phase ratio prevented the formation of a continuous C-S-H gel network. This is clearly observed in SEM images as wide voids, microcracks, and discontinuous gel structures. As stated in the literature, the addition of petroleum-derived wastes to cement-based systems leads to a decrease in mechanical performance due to increased porosity and poor bonding [58]. As a result, the balance between the reactive binder phase and the inert additives is considered the fundamental parameter determining the continuity and microstructural integrity of the matrix structure.

### 3.5. Surface Area Analysis (BET)

BET analysis was performed to characterize the pore structure of all alkali-activated slag mortar samples containing kapok fiber, PC and WTRP. As a result of the analysis, the specific surface area, total pore volume, and average pore diameter values were determined. In the first group of samples (KP-0, KP-0.5, KP-0.75 and KP-1), the effect of kapok fiber content ratio on pore structure was evaluated. SEM images revealed the formation of new void regions at the fiber–matrix interface and changes in matrix morphology with the addition of fibers. BET analysis was used to quantitatively evaluate these observations.

**Figure 11:**
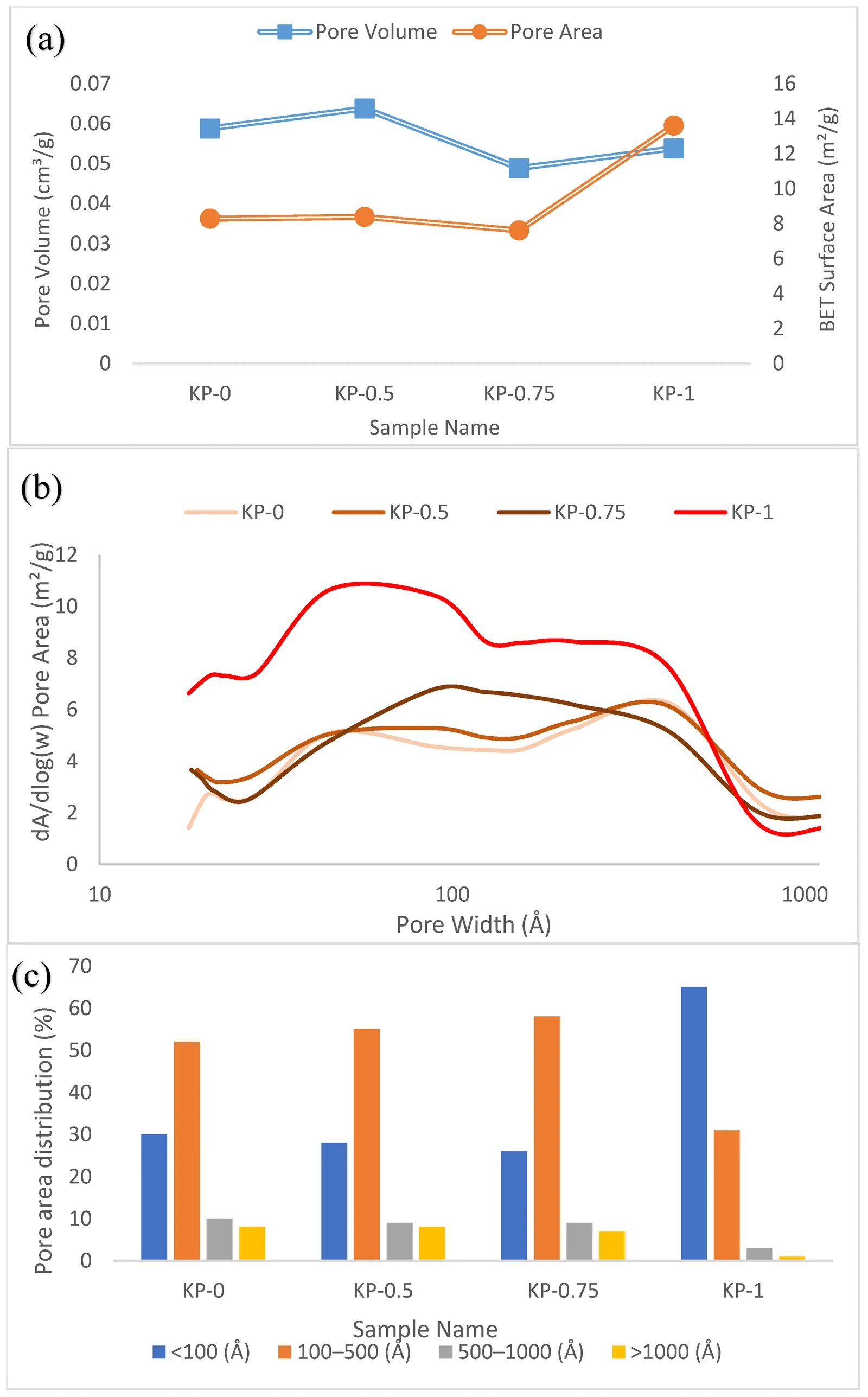
BET analysis of alkali-activated slag mortars incorporating kapok fiber. (**a**) BET surface area and total pore volume values of alkali-activated slag mortars containing kapok fiber. (**b**) Pore size distribution curves. (**c**) Pore area distribution as a function of pore width.

BET specific surface area and total pore volume values are given in Figure 11a,b, which show the pore size diameter distributions of the samples, while Figure 11c reveals the differential pore area distributions depending on the pore width. In general, the findings reveal that fiber addition has a dominant effect, especially for the 1.0% fiber addition, compared to the reference case. Compared to the reference sample, the total pore volume for KP-0.5, KP-0.75, and KP-1 samples shows a limited change (8.48–18.86%), which is in an expected range, considering the increase in fiber amount. On the other hand, while the total pore area values did not show a significant change for KP-0.5 and KP-0.75, a significant increase of 64.19% is detected for KP-1. In addition, while the average pore diameter in KP-0.5 and KP-0.75 samples shows a limited change (7.22–9.52%) compared to the KP-0 sample, a significant decrease in average diameter of 44.27% was also detected in the KP-1 sample, where a significant increase in total pore area was observed. Taken together, Figure 11b highlights the effect of fiber addition on the modified pore size distribution. From the results presented above, it is apparent that the dominance of smaller diameter pores in the KP-1 sample aligns with the significant decrease in average pore diameter and the increase in BET surface area. Despite this decrease in pore diameter, the fact that the total pore volume remains at levels close to the reference sample indicates the development of a structure consisting of finer and more numerous pores. This suggests that the increase in surface area is related to pore thinning and the formation of new interfaces rather than total porosity. While limited changes in pore volume were observed at low fiber ratios, the incorporation of 1% kapok fiber reduced the average pore diameter and significantly increased the specific surface area. Figure 11c indicates that the differential pore area distributions exhibit a change in pore area and volume distribution towards smaller diameter pores as the ratio of kapok fiber content increases. In the KP-1 sample, the contribution of pore diameters in the <100 Å range increased markedly, whereas the proportion of larger diameter pores diminished. This aligns with an increase in specific surface area without a substantial increase in total pore volume, suggesting that the incorporation of fibers generates a more refined and extensive internal pore network. The effectiveness of fiber reinforcement in cement mortars depends not only on fiber type and dosage but also on the characteristics of the surrounding matrix [66]. This consideration is particularly relevant to the present study, in which the matrix characteristics were progressively modified through the incorporation of petroleum coke and waste tire rubber powder. Therefore, the contribution of kapok fiber should be interpreted in relation to the evolving pore structure, interfacial characteristics, and mechanical and thermal behavior of these multicomponent matrices.

**Figure 12:**
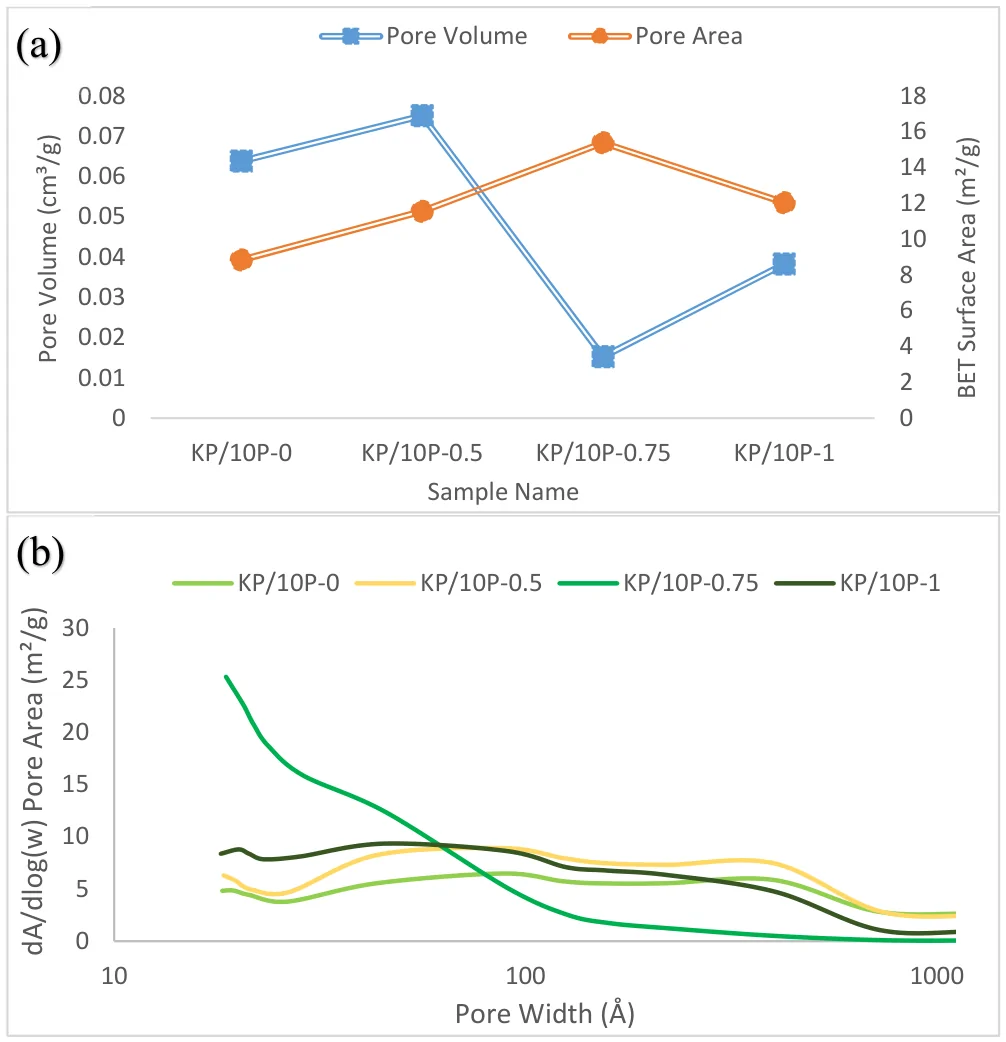

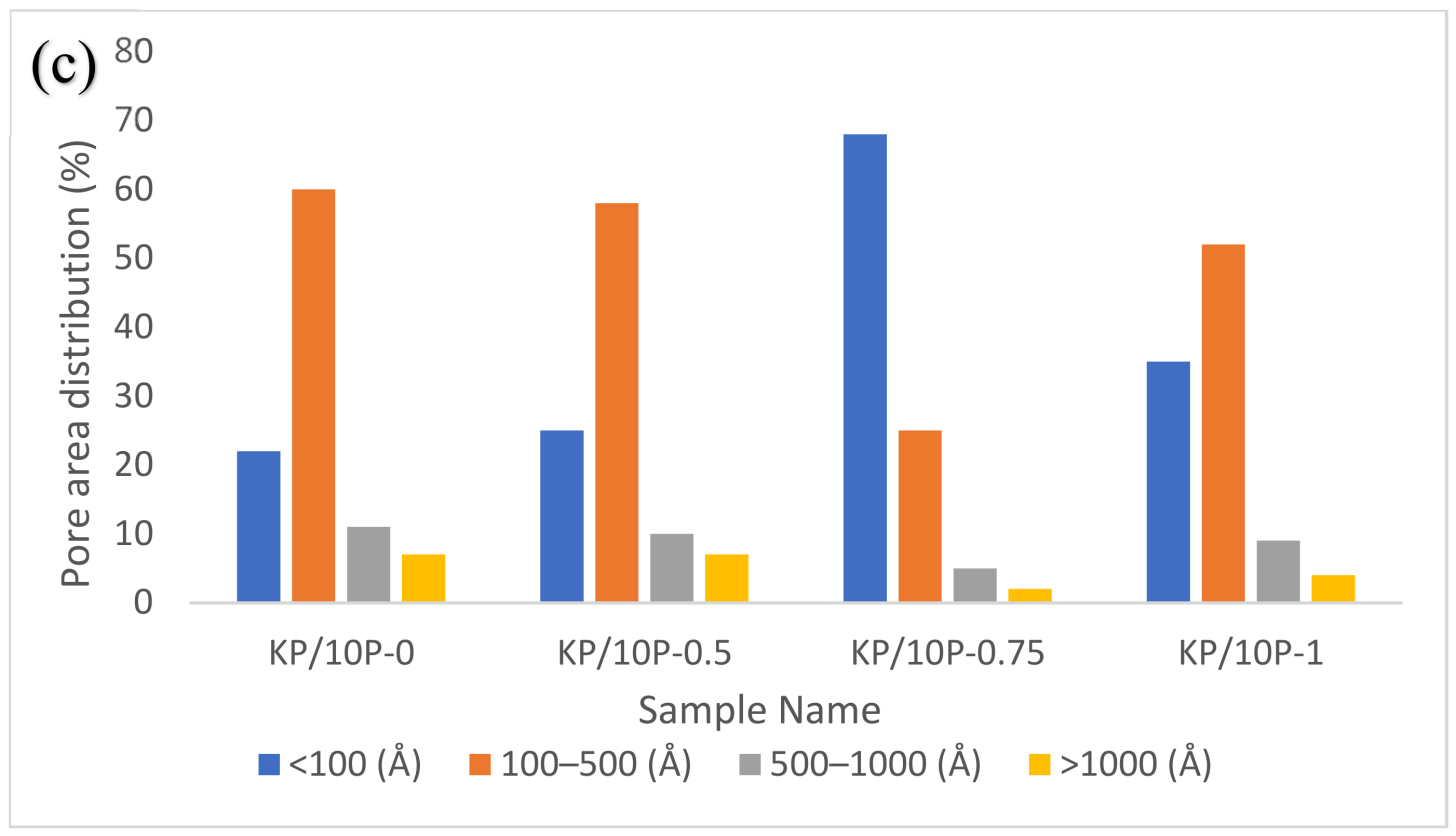
BET analysis of alkali-activated slag mortars incorporating kapok fiber and 10% PC. (**a**) BET surface area and total pore volume values of alkali-activated slag mortars containing kapok fiber and 10% PC. (**b**) Pore size distribution curves. (**c**) Pore area distribution as a function of pore width.

Figure 12a presents the BET specific surface area and total pore volume results of alkali-activated mortar samples containing different ratios of kapok fiber and 10% PC, while Figure 12b,c show the pore diameter distributions and the pore area distribution as a function of pore width, respectively. The average pore diameters were determined to be 288.86, 259.88, 39.67, and 127.07 Å in KP/10P-0, KP/10P-0.5, KP/10P-0.75, and KP/10P-1 samples, respectively. Increasing the kapok fiber content from 0% to 0.75% increased the BET surface area from 8.84 m^2^/g to 15.38 m^2^/g, while the total pore volume decreased from 0.0638 cm^3^/g to 0.0153 cm^3^/g. The decrease in average pore diameter also indicates that the pore structure has been refined. As shown in Figure 12b, the KP/10P-0.75 sample exhibited a distribution concentrated at smaller pore diameters, which is consistent with the lowest average pore diameter value. Conversely, with 1% fiber content, the surface area diminished to 12.04 m^2^/g while the pore volume increased once more. The measured BET values align with the microstructural modifications detected in SEM investigations resulting from fiber incorporation. Within groups comprising 10% PC, sample KP/10P-0.75 demonstrated the largest specific surface area, the lowest pore volume, and the smallest average pore diameter, indicating the most compact pore structure. Therefore, it is evaluated that the KP/10P-0.75 sample containing 0.75% kapok fiber exhibits the most suitable microstructural behavior by developing a finer and more homogeneous pore structure compared to the other samples and may represent an optimum ratio for kapok fiber addition in mixtures containing 10% PC. Figure 12c shows that the pore area distribution in the KP/10P group changes significantly with increasing fiber content. In the KP/10P-0 and KP/10P-0.5 samples, the most common pore area is found within the 100-500 Å range; however, in the KP/10P-0.75 sample, almost 68% of the area includes smaller diameter pores ranging less than 100 Å. This is consistent with the highest BET surface area (15.38 m^2^/g) and lowest average pore diameter (39.67 Å) values determined in the same sample. In the KP/10P-1 sample, the fraction of small-diameter pores diminished, and the distribution expanded throughout wider range of dimensions. The results show that a 0.75% kapok fiber content creates a more advanced internal surface structure by concentrating the pore surface in finer diameter pores compared to systems containing 10% PC.

**Figure 13:**
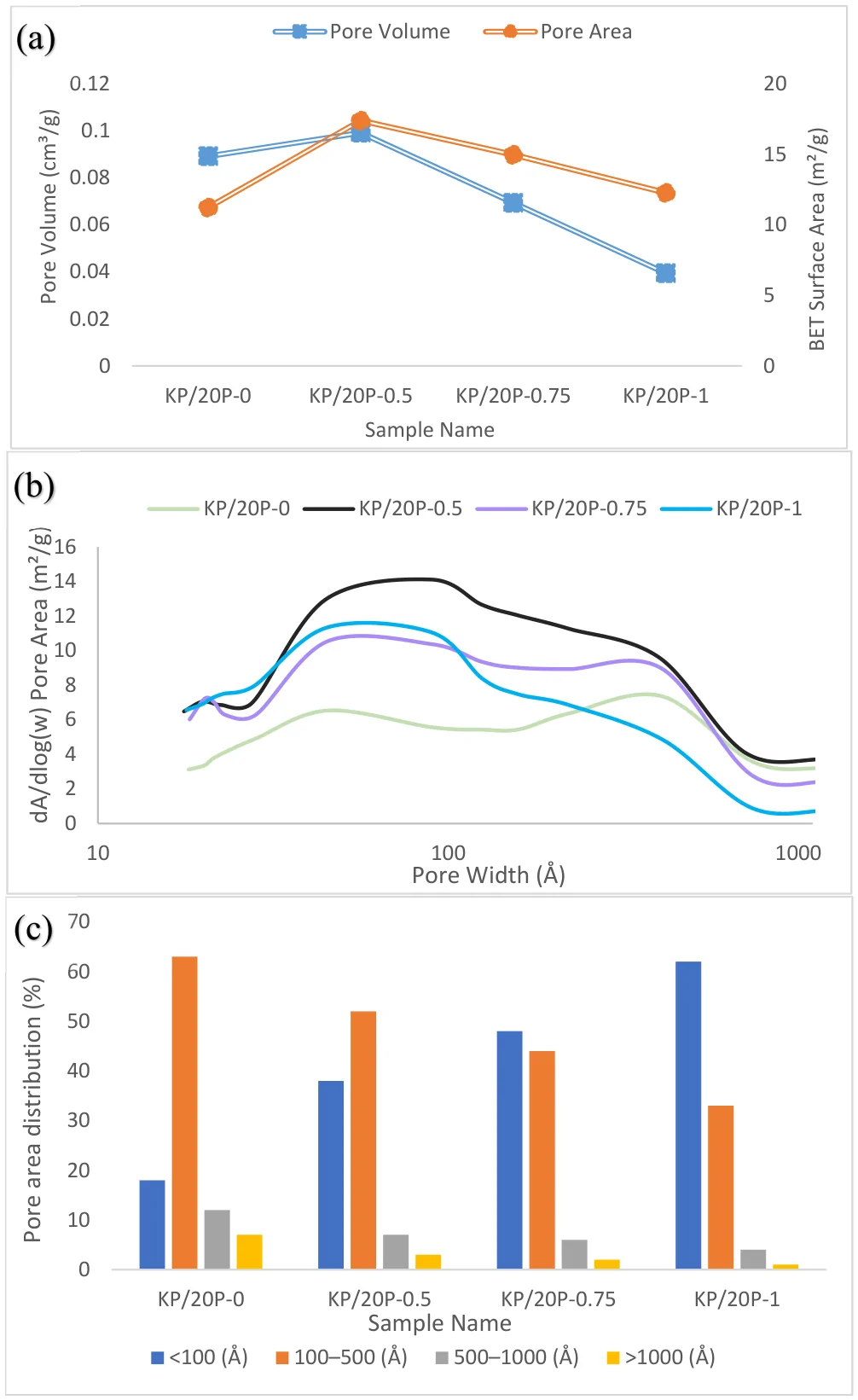
BET analysis of alkali-activated slag mortars incorporating kapok fiber and 20% PC. (**a**) BET surface area and total pore volume values of alkali-activated slag mortars containing kapok fiber and 20% PC. (**b**) Pore size distribution curves. (**c**) Pore area distribution as a function of pore width.

Figure 13a displays the BET specific surface area and total pore volume results of alkali-activated mortar samples containing different ratios of kapok fiber and 20% PC at different content ratios, while Figure 13b,c display the pore diameter distributions and the pore area distribution as a function of pore width, respectively. The average pore diameters were measured at 317.72, 228.16, 185.36 and 128.51 Å for the KP/20P-0, KP/20P-0.5, KP/20P-0.75, and KP/20P-1 samples, respectively. The addition of kapok fiber resulted in a noticeable reduction in pore size, with a consistent decrease in average pore diameter. The highest BET surface area and total pore volume were obtained in the KP/20P-0.5 sample, with values of 17.39 m^2^/g and 0.0992 cm^3^/g, respectively. With an increase in fiber content to 0.75% and 1%, the pore volume diminished to 0.0694 cm^3^/g and 0.0394 cm^3^/g, respectively, as well as a related reduction in average pore diameter. Figure 13b displays that the pore distribution changed to smaller diameter ranges in samples with high fiber content. Specifically, sample KP/20P-1 exhibited the lowest total pore volume and the smallest average pore diameter. The findings align with the denser and more continuous matrix structure identified in the KP/20P-1 sample in SEM analysis. When BET results and SEM observations are concurrently assessed, it can be concluded that increasing the kapok fiber ratio above 0.5% contributed to a significant refinement of the pore structure and led the formation of a more compact microstructure; however, the high fiber content made homogeneous fiber distribution difficult, limiting the effectiveness of microstructural improvement. Therefore, while the 0.5% kapok fiber ratio exhibited optimum behavior in terms of high internal surface area and pore development, higher fiber ratios, although tightening the pore structure, did not provide any predictable steady microstructural behavior. Figure 13c illustrates that the incorporation of kapok fiber results in a concentration of the pore surface in increasingly smaller diameter pores. In the KP/20P-0 sample, the majority of the pore area was within the 100–500 Å range, but the contribution of pores in the <100 Å range markedly increased with fiber content. In the KP/20P-0.5 sample, exhibiting the highest BET surface area, the fraction of small-diameter pores markedly increased. Conversely, in the KP/20P-1 sample, despite a reduction in total pore volume, the majority component of the pore area consisted of fine pores. This finding aligns with results indicating a reduction in average pore diameter and BET surface area, suggesting that kapok fiber enhances pore structure and increases surface area through a higher proportion of small pores, especially in systems with elevated PC content.

**Figure 14:**
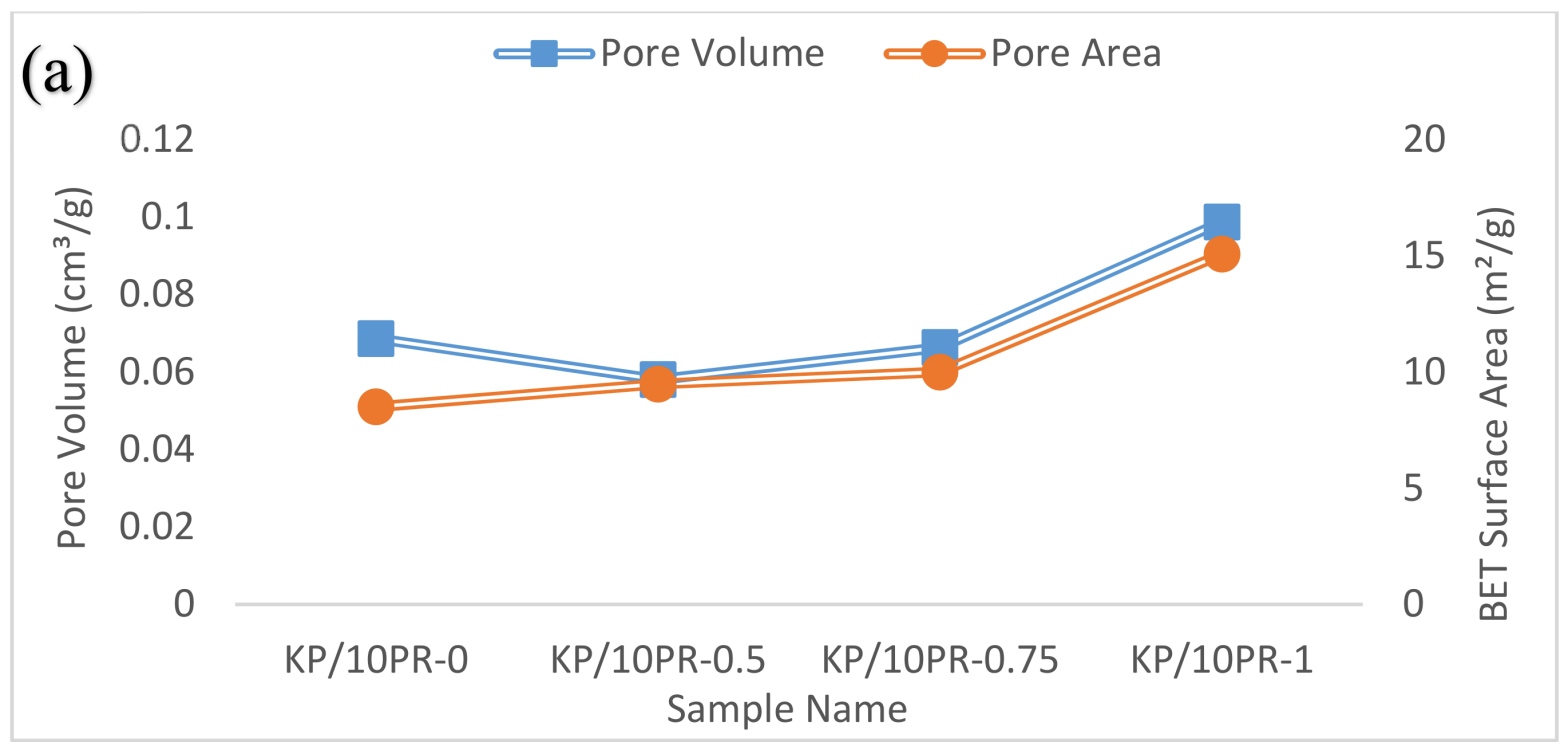

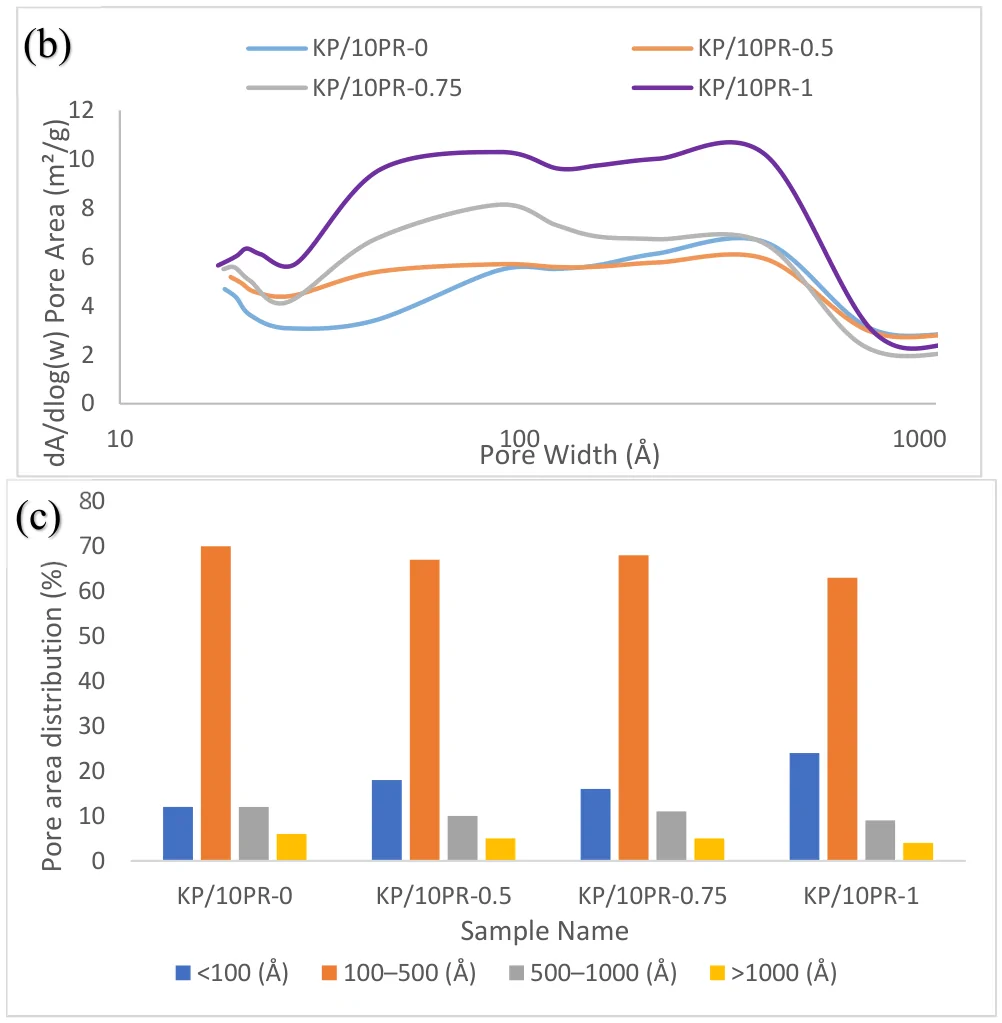
BET analysis of alkali-activated slag mortars incorporating kapok fiber, 10% PC and WTRP. (**a**) BET surface area and total pore volume values of alkali-activated slag mortars containing kapok fiber, 10% PC and WTRP. (**b**) Pore size distribution curves. (**c**) Pore area distribution as a function of pore width.

Figure 14a illustrates the BET specific surface area and total pore volume results of alkali-activated mortar samples containing kapok fiber, 10% PC, and 3% WTRP at different content ratios, while Figure 14b,c illustrate the pore diameter distributions and the pore area distribution as a function of pore width, respectively. The average pore diameters were determined to be 322.93, 244.84, 265.35 and 262.11 Å in the KP/10PR-0, KP/10PR-0.5, KP/10PR-0.75 and KP/10PR-1 samples, respectively. An overall increase in specific surface area was observed with the addition of kapok fiber. The lowest BET surface area was determined in the KP/10PR-0 sample with 8.51 m^2^/g, while the highest value was obtained in the KP/10PR-1 sample with 15.07 m^2^/g. In parallel, the total pore volume also reached a value of 0.0988 cm^3^/g in the KP/10PR-1 sample. Increasing the kapok fiber ratio to 0.5% reduced the average pore diameter to 244.84 Å, but both the specific surface area and the total pore volume increased again at higher fiber ratios. The pore distribution curves in Figure 14b show that a wider pore network is formed with the addition of fibers, and that the number of pores increases in different diameter ranges, especially in the KP/10PR-1 sample. These findings are consistent with the fiber network structure, perifiber voids, and heterogeneous matrix regions observed in the KP/10PR-1 sample in SEM analysis. The results indicated that the interaction between the hydrophobic nature of WTRP and the distribution of kapok fibers within the matrix, along with the high fiber content, creates a more complex and heterogeneous pore network. In other words, the internal surface area in the KP/10PR-1 sample increased significantly due to the formation of numerous pores, but this may be due to additional voids formed at the fiber–matrix and tire–matrix interfaces rather than a refinement of the pore structure. Therefore, it can be said that in the series containing 10% PC and 3% WTRP, the high kapok fiber content, after a certain level, leads to the formation of a more heterogeneous and porous microstructure, rather than improving the pore structure. In this respect, while the KP/10PR-0.5 and KP/10PR-0.75 samples exhibit a more balanced pore structure, the KP/10PR-1 sample shows a structural behavior where interfacial voids become dominant due to the high fiber content. Examining the pore area distributions of the KP/10PR samples given in Figure 14c, it is observed that the majority of the pore area in all samples is concentrated in the 100-500 Å range. In the mixture containing 10% PC and 3% WTRP, a significant increase in BET surface area was observed with increasing kapok fiber content, with the highest value obtained in the KP/10PR-1 sample. At the same time, the total pore volume also increases, reaching 0.0988 cm^3^/g. The porous, low-density structure of PC, combined with the interstitial voids produced by WTRP and the supplementary voids generated by kapok fibers, has resulted in a more complicated pore network. The increase in the contribution of small and medium diameter pores to the surface area in the pore area distributions indicates that a more accessible inner surface is formed within the matrix. The findings indicate that the synergistic application of PC, WTRP and kapok fiber markedly modifies the microstructure, enhancing both the specific surface area and the total pore volume. This trend aligns with the heterogeneous matrix structure and voids surrounding the fibers found in SEM images, suggesting that the extra interfaces introduced by PC and WTRP are reflected in the BET results.

**Figure 15:**
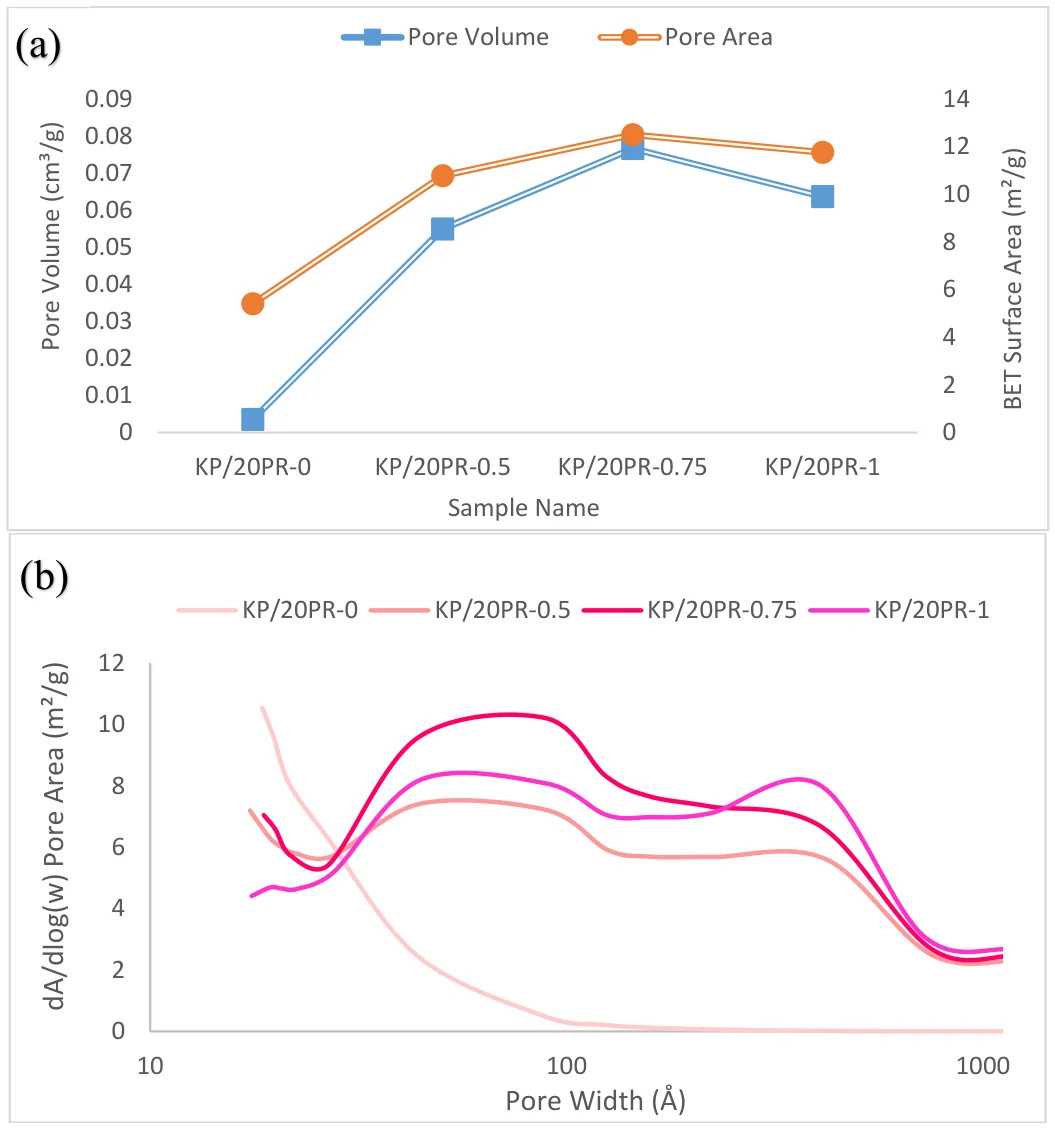

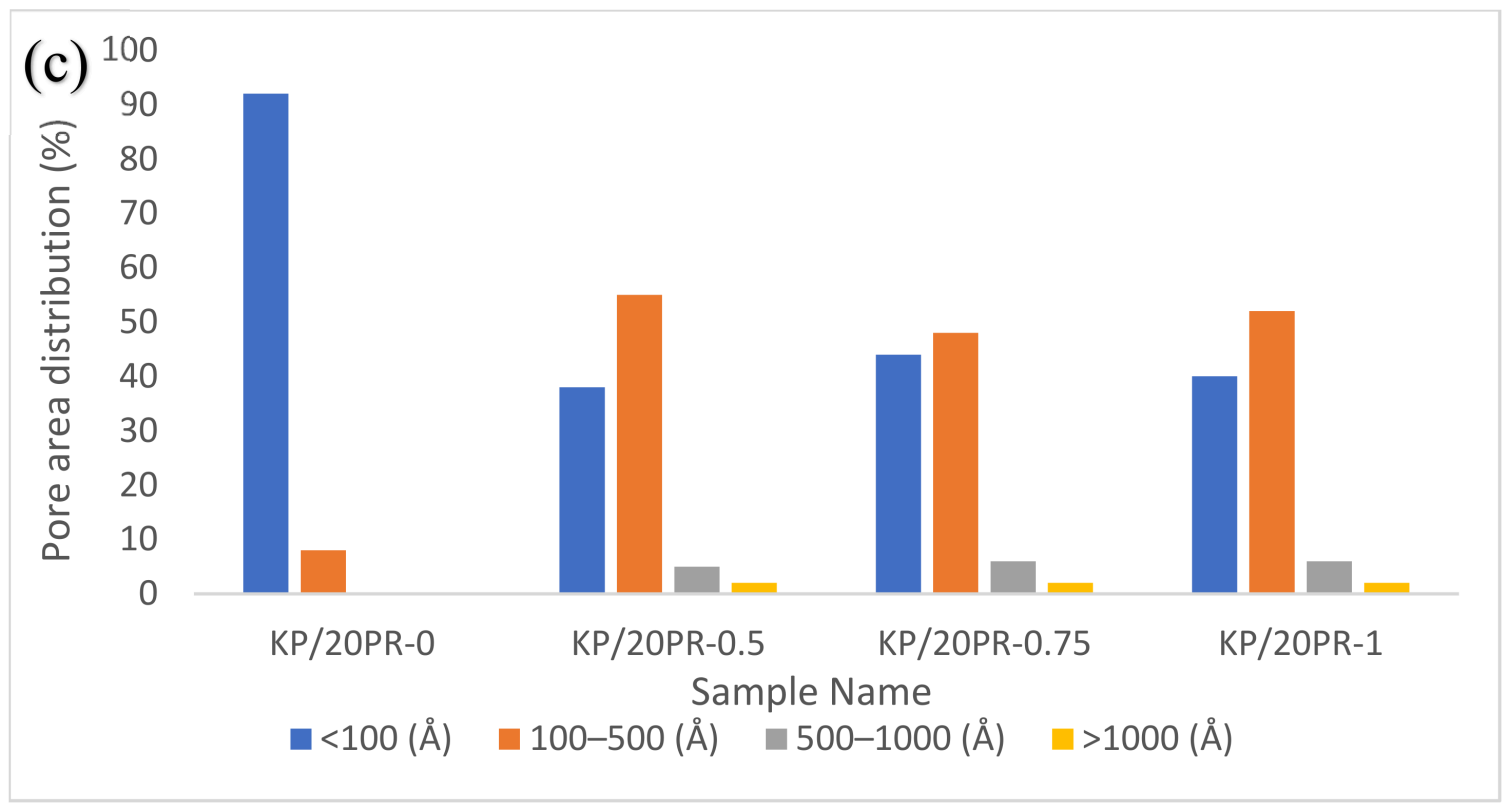
BET analysis of alkali-activated slag mortars incorporating kapok fiber, 20% PC and WTRP. (**a**) BET surface area and total pore volume values of alkali-activated slag mortars containing kapok fiber, 20% PC and WTRP. (**b**) Pore size distribution curves. (**c**) Pore area distribution as a function of pore width.

Figure 15a demonstrates the BET specific surface area and total pore volume outcomes of alkali-activated mortar samples incorporating different ratios of kapok fiber, with 20% PC and 3% WTRP additions, while Figure 15b,c demonstrate the pore diameter distributions and the pore area distribution as a function of pore width, respectively. The average pore diameters were measured at 26.22, 203.91, 245.21, and 216.60 Å for the KP/20PR-0, KP/20PR-0.5, KP/20PR-0.75 and KP/20PR-1 samples, respectively. The KP/20PR-0 sample without kapok fiber exhibited the most compact structure, with a BET surface area of 5.41 m^2^/g and a total pore volume of 0.0035 cm^3^/g. The incorporation of kapok fiber led to a significant enhancement in both the specific surface area and the total pore volume. The highest BET surface area of 12.50 m^2^/g and the highest total pore volume of 0.0766 cm^3^/g were obtained in the KP/20PR-0.75 sample. Figure 15b illustrates that the incorporation of kapok fiber results in an expansion of the pore structure to a wider diameter range, as well as an increase in the quantity of pores contributing to the overall pore volume. The notable increase in average pore diameter in KP/20PR-0.5 and KP/20PR-0.75 samples suggests that kapok fibers generate supplementary voids inside the matrix of PC and WTRP. Although a partial decrease in surface area and pore volume was observed when the fiber content was increased to 1%, the values remained at a higher level compared to the reference sample. The obtained BET results align with the fiber network structure, the interval voids around the fibers, and heterogeneous matrix regions identified in the SEM investigation of the KP/20PR-1 sample. The SEM images indicate that the incorporation of fibers generates new interfaces and voids within the matrix, whereas BET measurements quantitatively confirm this effect through an increase in specific surface area and total pore volume. In conclusion, the synergistic application of kapok fiber, PC and WTRP markedly modified the pore structure, facilitating the development of a more sophisticated and accessible pore network. Figure 15c illustrates that the incorporation of kapok fiber markedly modifies the pore area distributions in mixes including 20% PC and 3% WTRP. In the P/20PR-0 sample, the majority of pore area is detected in small-diameter pores that are below 100 Å, whereas the incorporation of kapok fiber expands the pore area to a larger diameter range. In KP/20PR-0.75 and KP/20PR-1 samples, the contribution of pores in the 100-500 Å range to the surface area increased significantly. The KP/20PR-0.75 sample, exhibiting the highest BET surface area, demonstrated a more balanced distribution, with both small- and medium-sized pores contributing to the overall surface area. This situation demonstrates that the heterogeneous composition of PC and WTRP, together with kapok fibers, creates a more intricate and accessible internal surface. The results reveal that the addition of fibers not only increases the pore area but also alters the dimensional distribution of the pores, creating a more advanced pore network. The evaluation of BET results with SEM observations suggests that the heterogeneous matrix region created by fiber incorporation and voids at the fiber–matrix interface contribute to this behavior.

BET results indicated that the incorporation of kapok fiber substantially modified the microstructural properties of the mortars. Modifications in specific surface area, total pore volume, and average pore diameter were noted based on the combination composition, with a more significant impact in mixtures incorporating PC and WTRP. While a more developed pore network was observed in samples containing 0.75–1.0% kapok fiber, some mixtures showed limited reductions in pore parameters due to high fiber ratios. Similarly, it has been reported in the literature that natural fibers rearrange the pore structure in alkali-activated systems, create new voids at the fiber–matrix interface, and significantly affect BET parameters [67]. Furthermore, it has been noted that the multi-scale void network formed by plant fibers is related to the increase in specific surface area and changes in pore structure [68]. The results indicate that kapok fiber modifies the pore structure by generating new interfaces and void regions within the matrix, significantly influencing the microstructural properties of the mortars. Furthermore, the changes in pore structure determined in BET analysis are consistent with the thermal conductivity results obtained in the samples. In particular, the lower thermal conductivity values observed in mixtures with more advanced and heterogeneous pore structures support the effect of the pore network on the heat transfer mechanism.

### 3.6. X-Ray Diffraction (XRD) Analysis

**Figure 16:**
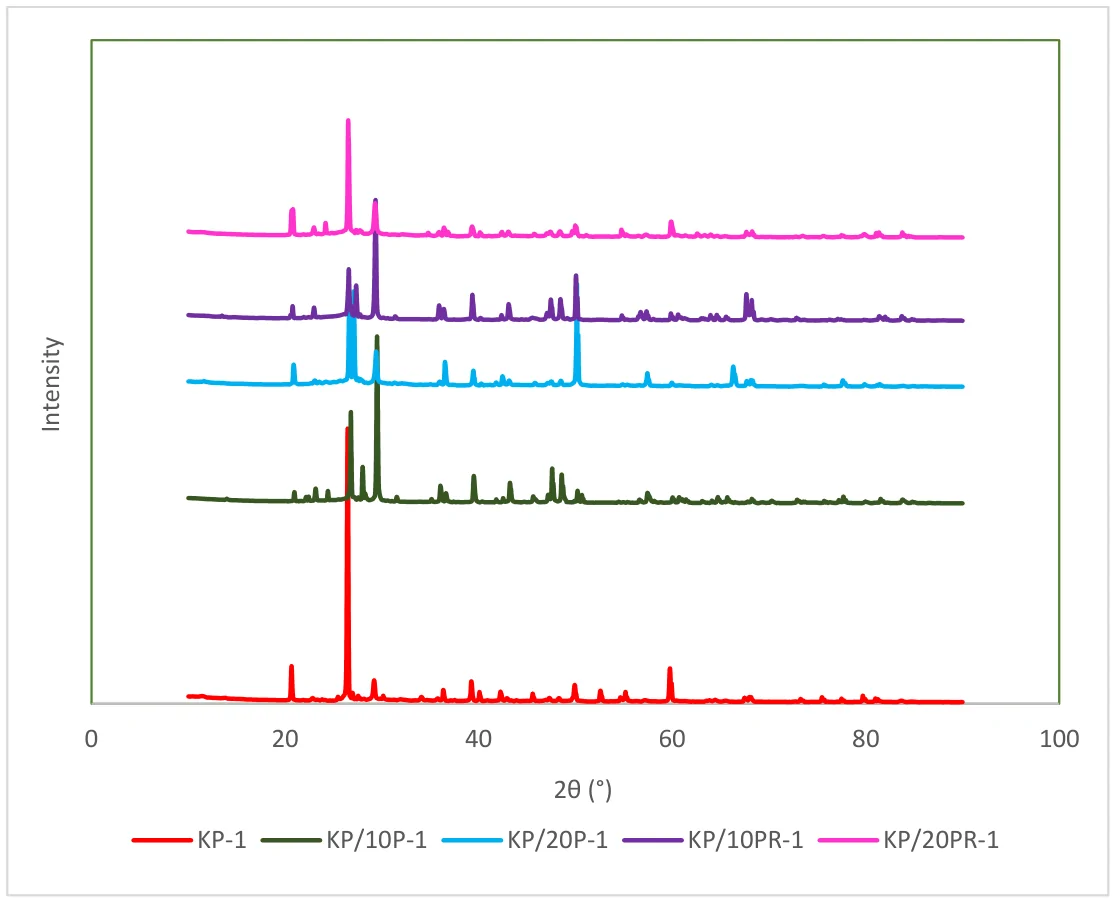
X-ray diffraction results of fiber incorporated mortar samples.

X-ray diffraction (XRD) analysis was performed to determine the mineralogical composition of mortar samples and the effect of additives on hydration products. XRD analysis was applied not to the entire mixture, but only to selected samples showing the highest insulation performance in terms of thermal conductivity results. Because the increase in PC and WTRP content within the same additive group creates similar reaction mechanisms, it was assessed that mineralogical changes would be most pronounced in samples with the highest additive ratios. Figure 16 shows the XRD patterns of selected mortar samples, and the characteristic peaks were evaluated to determine the phases formed. As a result of the hydration and reaction processes, it was observed that new phases formed in the binder system in addition to the phases initially present in the raw materials. The observed mineralogical changes are thought to be more influenced by the quantity of PC and WTRP incorporated into the mixture than by the inclusion of fiber. The literature indicates that carbon-based inert additives, such as PC, can diminish C-S-H formation and enhance porosity by reducing the binder phase [64]. Similarly, it has been reported that the hydrophobic nature of WTRP can limit the development of hydration products and negatively affect matrix continuity [13,15]. The use of slag not only contributes to the circular economy by reducing clinker dependency and associated environmental impacts, but also acts as an active binder constituent that influences mechanical performance and pore structure through the formation of hydration products. The pozzolanic and latent hydraulic reactions of slag can generate additional C-S-H gel, thereby contributing to the development of the cementitious matrix and refinement of its pore structure [69].

In the XRD patterns, the sharp peaks observed around 2θ ≈ 26.6°, 50° and 59–60° in the KP-1 sample correspond to the quartz (Q) phase and originate from the aggregate. In contrast, the broad diffuse peak observed in the 20–32° range represents the formation of the binder gel phase, C-S-H, while the peak around 2θ ≈ 34–35° represents the portlandite (CH) phase. This phase distribution is consistent with the typical microstructure reported in cement-based and alkali-activated systems [70].

The XRD pattern of the KP/10P-1 sample exhibited a prominent quartz (Q) peak at 2θ = 26.6°, indicating the main crystalline phase. Moreover, it is associated with the formation of the amorphous region-binding gel phase, C-S-H, which displays a broad distribution in the 20–32° range. The weak peak observed around 2θ ≈ 34–35° corresponds to the portlandite (CH) phase. Secondary peaks associated with the quartz phase were additionally observed at around 50° and 59–60°. Regarding the inert and low-reactivity characteristics of PC addition, it is evaluated that this additive may indirectly affect the formation of hydration products within the binder system rather than directly increasing them. This finding aligns with the mechanical strength results obtained.

The XRD pattern of the KP/20P-1 sample displayed a prominent quartz (Q) peak at 2θ = 26.6°, indicating the main crystalline phase. Furthermore, the amorphous region, with a broad distribution through the 20–32° range, has been associated with the formation of the C-S-H binding gel phase. The peak observed around 2θ ≈ 34–35° corresponds to the portlandite (CH) phase. Secondary peaks associated with the quartz phase were also detected at around 50° and between 57° and 60°. This sample has a lower relative contribution of the amorphous gel phase compared to previous groups, although the portlandite phase is comparatively more prominent. This suggests that the increasing PC content may have a limiting effect on the development of hydration/activation products within the binder system, which is consistent with the obtained mechanical strength results.

The XRD pattern of the KP/10PR-1 sample revealed a prominent quartz (Q) peak at 2θ = 26.6°, indicating it as the dominant phase. The broad amorphous region located in the 20–32° range is associated with the formation of the C-S-H binding gel phase, and the relative contribution of this phase is considered to be lower compared to the previous groups. It has been observed that the portlandite (CH) peak, which is seen around 2θ ≈ 34–35°, has become relatively more prominent. This suggests that the development of hydration/activation products may be limited in the binding system. The lack of new crystalline phase development in this system, utilizing PC and WTRP simultaneously, indicates that these additives are inert and may produce filling and interfacial effects inside the binder matrix. The findings align with the literature trend, indicating that a reduction in C-S-H formation and a corresponding increase in the CH phase can adversely impact compressive strength [64].

The XRD pattern of the KP/20PR-1 sample exhibits a prominent quartz (Q) peak at 2θ = 26.6°, indicating the main crystalline phase. The broad amorphous region located in the 20–32° range has been associated with the formation of the binding gel phase, C-S-H. However, the relative contribution of this gel phase is considered to be lower compared to previous groups. The peak observed around 2θ ≈ 34–35° corresponds to the portlandite (CH) phase. Secondary peaks belonging to the quartz phase were also identified around 50° and 59–60°. These findings suggest that the formation of the binder gel may have been limited due to the high inert admixture content, while the portlandite phase became relatively more prominent. This is considered to exhibit a trend consistent with the observed decrease in mechanical strength.

The XRD findings obtained in this study reveal a microstructural trend consistent with the compressive strength results. While the increase in the C-S-H gel phase formed during the hydration process improves strength by increasing matrix continuity, the relative increase in the free portlandite (CH) phase can reduce binder effectiveness and lead to mechanical losses. Indeed, the fact that the compressive strength values in the KP-1 and KP/10P-1 samples are very close to each other (36.15 and 35.88 MPa) can be explained by the preservation of a similar phase distribution in XRD analysis and only a limited increase in CH. In contrast, the lower strength values (30.1 and 29.59 MPa) in KP/20P-1 and KP/20PR-1 samples are associated with the relative decrease in the C-S-H gel phase and the more prominent CH phase. This indicates that an increased proportion of inert additives may restrict the development of hydration products. The literature indicates that enhanced formation of C-S-H gel correlates with increased mechanical strength, whereas an increase in the CH phase may adversely impact strength [64,70]. It is also known that inert additives such as PC and WTRP can limit binder gel development by creating discontinuities in the matrix. Similarly, in this study, phase changes observed, especially at high additive content ratios, are considered one of the main causes of mechanical losses. The literature indicates that, regarding fiber reinforcing, fibers do not directly create a new crystalline phase; instead, they enhance mechanical performance through a crack-bridging mechanism [65,71].

It is worth repeating that the KP-1 mortar exhibited approximately 20.7–28.5% higher thermal conductivity value compared to the samples containing different amount of PC and waste rubber at 1.0% fiber content. As discussed previously, the reduction in thermal conductivity associated with PC incorporation is mainly attributed to its amorphous and porous microstructure. The non-crystalline nature and internal closed pores of PC restrict heat transfer and thermal wave propagation, while its hydrophobic behavior promotes air entrainment and additional void formation within the matrix. Moreover, the inherently low density of PC contributes to increased porosity, further enhancing the thermal insulation performance of the KP/10P-1, KP/20P-1, KP/10PR-1, and KP/20PR-1 samples [58]. On the other hand, the incorporation of waste rubber particles reduces mechanical strength due to their low elastic modulus, hydrophobic nature, and weak ITZ formation with the cementitious matrix, which increase porosity and disrupt stress transfer continuity. Additionally, the resulting heterogeneous pore structure and entrapped air voids may contribute to lower thermal conductivity by limiting heat transfer within the matrix [72]. XRD findings showed a trend consistent with pore structure changes identified in BET analysis and microstructural discontinuities observed in SEM images. The observed reduction in the C-S-H gel phase, particularly in mixtures incorporating PC and WTRP, was assessed alongside alterations in pore volume and surface area identified through BET analysis, as well as the more heterogeneous matrix structure and void formations noted in SEM images. The mechanism behind the thermal conductivity and compressive strength reductions induced by PC and WTRP can clearly be observed in the XRD patterns of the samples, in agreement with the CH, C-S-H, and Q peaks, as explained in detail above.

### 3.7. Thermogravimetry and Differential Scanning Calorimetry (TG-DSC) Analysis

**Figure 17:**
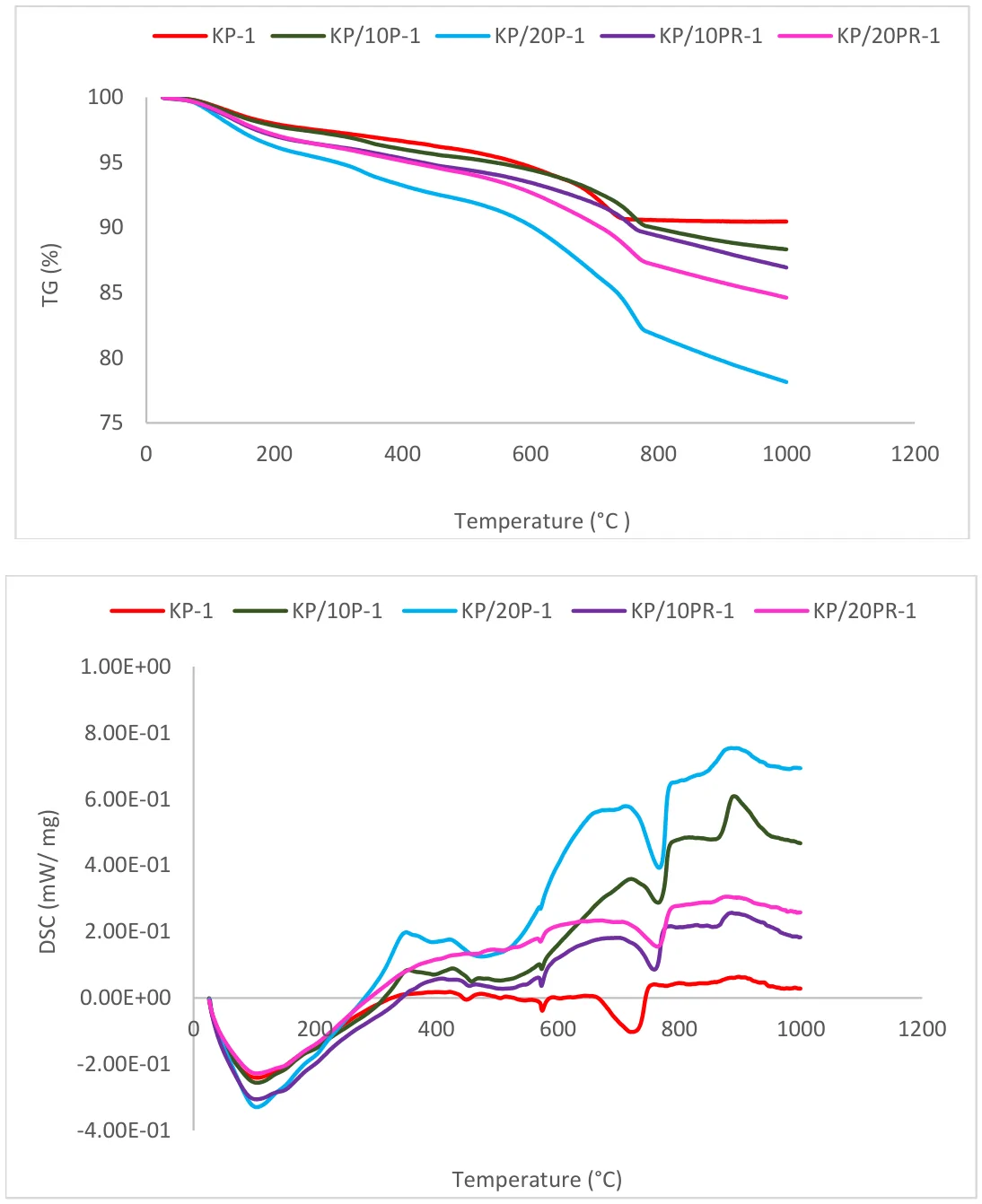
TG-DSC results of fiber incorporated mortar samples.

Thermogravimetric (TG) and differential scanning calorimetry (DSC) analysis was performed to determine the thermal stability and temperature-dependent degradation behavior of mortar samples. Analyses were conducted on samples KP-1, KP/10P-1, KP/20P-1, KP/10PR-1 and KP/20PR-1, which exhibited the highest insulating performance within each mixture group, based on thermal conductivity results. Figure 17 shows the TG-DSC curves for these mortar samples. All samples exhibited similar thermal decomposition behavior, with initial mass loss occurring within the temperature range of around 50–200 °C. All samples exhibited similar thermal degradation behavior, with initial mass loss occurring within the temperature range of around 50–200 °C. Losses in this region can be attributed to the elimination of physically bonded water and water retained within hydration products. As temperature increases, mass losses become more constrained in the 200–600 °C range, attributable to the progressive decomposition of alkali-activated binder phases and the thermal degradation of organic components in the mixture. The second distinct region of change observed in the 650–800 °C range can be attributed to the degradation of more stable reaction products within the binding system and phases that decompose at high temperatures. Moreover, it was noted that in all samples, mass loss did not entirely complete after 800 °C and persisted at a low trend until 1000 °C. This indicates the existence of more stable phases and residual components in the system that continue to degrade at higher temperatures.

When the samples were evaluated individually, sample KP-1 showed a more distinct C-S-H gel phase in XRD analyses, a more continuous matrix structure in SEM images, and a more stable pore structure in BET analyses. This aligns with elevated compressive strength and comparatively low total mass loss. Despite the addition of PC in the KP/10P-1 sample, a similar phase distribution was maintained in XRD analyses, and compressive strength values were found to be close to those of KP-1. In BET analysis, changes in pore structure and a reduction in thermal conductivity can be ascribed to the porous characteristics of PC. In the KP/20P-1 sample, the relative decrease in the C-S-H gel phase, the increased prominence of the portlandite (CH) phase, the heterogeneous matrix structure observed in the SEM images, and the changes in pore structure determined in the BET analyses were evaluated together. This sample showed lower compressive strength, lower thermal conductivity and higher overall mass loss. The KP/10PR-1 sample exhibited a high specific surface area and pore volume in BET analysis due to the synergistic application of PC and WTRP, whereas SEM pictures demonstrated perifiber voids and heterogeneous regions. No new crystalline phases were identified in the XRD investigations, and it is thought that the reduction in thermal conductivity and the mass loss observed in the TG curve are primarily attributable to alterations in the pore structure. In the KP/20PR-1 sample, XRD analysis revealed a relative decrease in the binder gel phase, SEM images showed more prominent void regions, and BET analysis revealed an improved pore network. This is consistent with low thermal conductivity values and also explains the decrease in compressive strength. The continued mass loss behavior at high temperatures in the TG curve also shows that this more heterogeneous and porous structure is reflected in the thermal decomposition process. Overall, the combined evaluation of SEM, BET, XRD, and TG-DSC data indicates that kapok fiber, PC and WTRP influence both mechanical and thermal performance by modifying the microstructure, pore characteristics, and phase distribution of alkali-activated mortars.

### 3.8. Optimization of Bio-Based and Waste-Derived Contents in Terms of Desired Mechanical-İnsulation Performance

**Figure 18:**
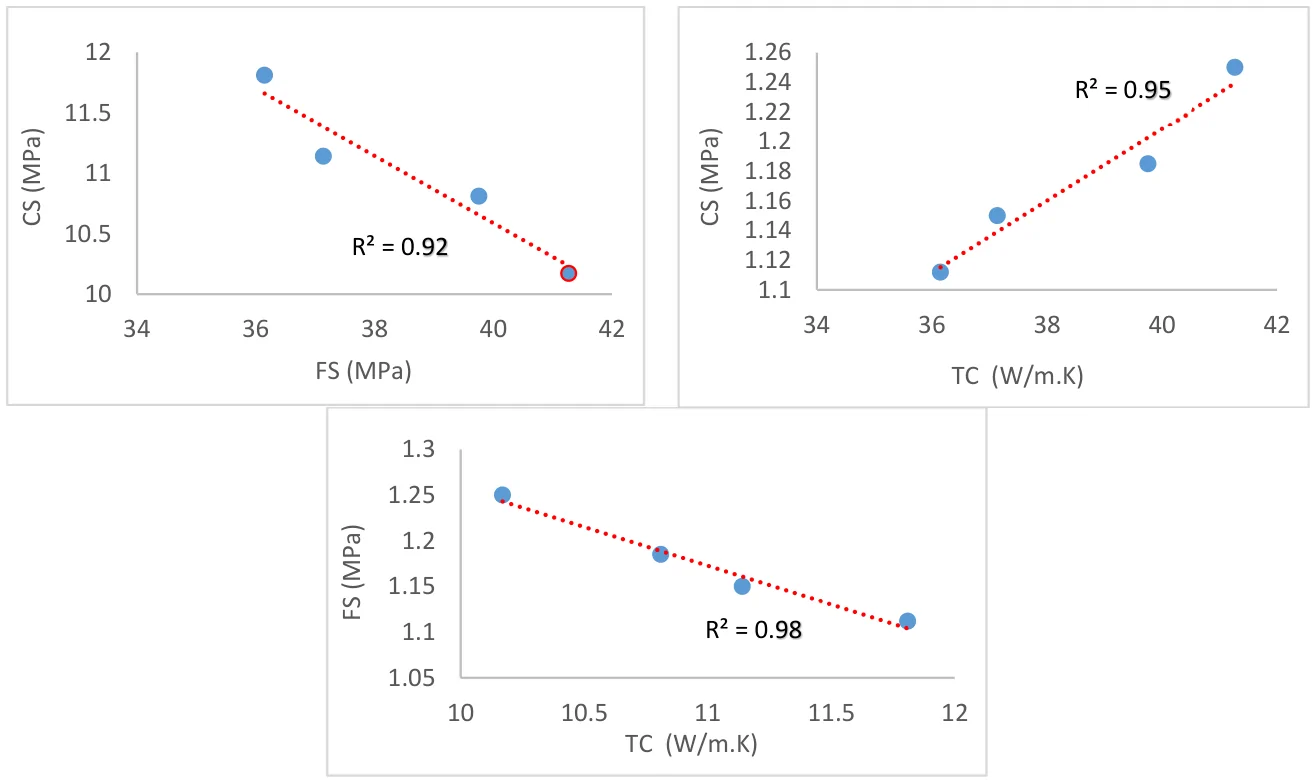
Correlations between mechanical strength and thermal conductivity in KP group.

**Figure 19:**
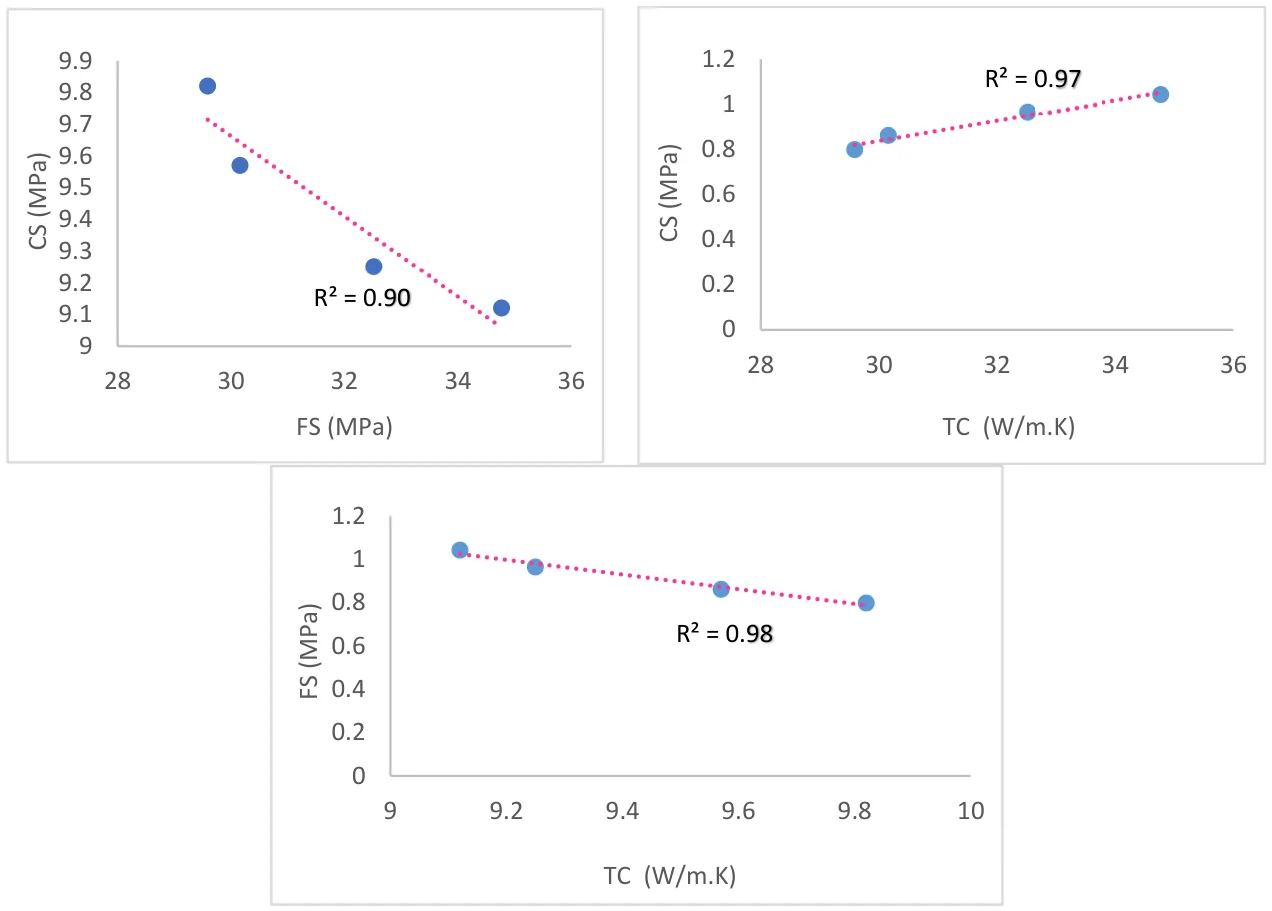
Correlations between mechanical strength and thermal conductivity in KP/20PR group.

It is a well known fact that mechanical performance and thermal conductivity behavior in cement-based composites are closely related to each other due to the dominant role of porous and microstructural characteristics on material properties. Therefore, evaluating these performance parameters together constitutes an important approach for optimum material design [51]. Based on this, linear correlations between compressive strength (CS), flexural strength (FS), and thermal conductivity (TC) values were evaluated to reveal the synergistic mechanism of bio-based and waste-derived materials on material properties. As is known, analyzing mechanical properties and thermal insulation performance together is considered an effective approach in optimizing the performance of sustainable mortar designs [19]. Figure 18 and Figure 19 illustrate the linear correlations between the reference (KP) and the KP/20PR groups, where the lowest and highest insulating performance were detected in KP and KP/20PR groups, respectively. This approach clearly demonstrates a general strategy for optimizing mechanical and thermal properties of sustainable mortar design.

The experimental findings revealed compressive strength values between 29.59 and 41.27 MPa, flexural strength values ranging from 9.12 to 11.82 MPa, and thermal conductivity values from 0.79 to 1.25 W/m·K. A significant interaction between the incorporation of kapok fiber and mechanical and thermal performance is revealed while analyzing the reference group (KP). The KP-0 sample, without fiber reinforcing, exhibited a compressive strength of 41.27 MPa, a flexural strength of 10.17 MPa, and a thermal conductivity of 1.25 W/m·K. Increasing the kapok fiber content to 1% enhanced thermal conductivity by 11.04%, reducing it from 1.25 W/m·K to 1.11 W/m·K, while simultaneously increasing flexural strength from 10.17 MPa to 11.81 MPa, and conversely diminishing compressive strength from 41.27 MPa to 36.15 MPa. This indicates that strong linear relationships between performance parameters are maintained throughout the increasing kapok fiber content. Strong correlation coefficients were observed between compressive strength and flexural strength (R^2^ = 0.92), compressive strength and thermal conductivity (R^2^ = 0.95), as well as flexural strength and thermal conductivity (R^2^ = 0.98).

Upon examination of the KP/20PR group, which has superior insulation performance, it is noted that the linear correlation between mechanical and thermal performance is maintained with the incorporation of kapok fiber. The KP/20PR-0 sample, without fiber reinforcing, exhibited a compressive strength of 34.77 MPa, a flexural strength of 9.12 MPa, and a thermal conductivity of 1.04 W/m·K. Increasing the kapok fiber content to 1% enhanced thermal conductivity by 23.41%, reducing it from 1.04 W/m·K to 0.79 W/m·K, while simultaneously increasing flexural strength from 9.12 MPa to 9.82 MPa, and conversely diminishing compressive strength from 34.77 MPa to 29.59 MPa. The results reveal that the synergistic effect of kapok fiber, 20% PC, and 3% WTRP significantly changes the pore structure and demonstrates a strong correlation between mechanical strength and thermal insulation performance. This reveals that the linear relationship between performance parameters is maintained throughout the increasing kapok fiber content. Significant correlation coefficients were achieved between compressive strength and flexural strength (R^2^ = 0.90), compressive strength and thermal conductivity (R^2^ = 0.97), and flexural strength and thermal conductivity (R^2^ = 0.98). The strong correlations detected highlight that the identified relationships are not only coincidental but reflect meaningful interactions that can provide a basis for material optimization.

Another important consideration is that modern design codes specify a minimum compressive strength of 25 MPa for structural concrete used in regions of high seismic hazard to enhance resistance to earthquake-induced effects [73]. The 28-day compressive strengths of all mixtures investigated in this study ranged from 29.59 to 41.27 MPa and thus remained above this minimum threshold. These results indicate that the developed mortars have potential for structural applications from the perspective of compressive strength. Nevertheless, compliance with the minimum compressive-strength requirement alone is insufficient to establish their overall structural suitability; long-term durability, full-scale structural behavior, and field performance should be further evaluated in future studies.

To further verify the reliability of the reported linear relationships, ANOVA was conducted for each regression model, and the results are summarized in Table 4. The ANOVA results confirmed the statistical significance of the suggested linear relationships, with high coefficients of determination (R^2^ = 0.878–0.982) and statistically significant regression models for the great majority of the investigated correlations (*p* < 0.05). Particularly, the relationships between flexural strength and thermal conductivity consistently displayed the highest R^2^ values (0.971–0.982), demonstrating a strong linear association between these performance parameters. Overall, the ANOVA results support the reliability of the proposed regression models and further confirm that the synergistic interaction of kapok fiber, PC and WTRP consistently governs both the mechanical and thermal performance of the developed mortar system. The strong correlations demonstrate that the interactions among the microstructural and pore-phase modifications introduced by kapok fiber, PC and WTRP affect both the mechanical and thermal properties via a common synergistic mechanism. Moreover, when interpreted in conjunction with the SEM, XRD, and BET results, these high correlation coefficients clearly confirm the existence of this synergistic mechanism. Although the synergistic mechanism was elucidated by examining the incremental effects of the individual constituents on thermal conductivity and mechanical performance, the heterogeneous cementitious system should ultimately be evaluated using a holistic approach. Increases in pore volume and specific surface area may reduce thermal conductivity by disrupting continuous heat-conduction pathways within the solid phase. However, the continued reduction in thermal conductivity despite the decreased pore volume in the series containing 20% PC indicates that thermal performance is not governed solely by total pore volume. An increased specific surface area accompanied by a reduced average pore diameter suggests the development of a finer, more complex, and tortuous pore network. Increased porosity and weakened particle–matrix interfacial regions may adversely affect compressive strength by reducing the effective load-bearing area and compromising matrix integrity. In contrast, the consistent improvement in flexural performance with increasing kapok fiber dosage across all experimental series indicates that fiber-induced crack bridging and stress transfer partially compensate for the adverse mechanical effects associated with the porous structure. Similarly, the continuous reduction in thermal conductivity with increasing kapok fiber content across all mixture groups demonstrates that heat transfer is collectively governed by pore volume, specific surface area, pore size, and heat-path tortuosity. Therefore, in cement-based systems developed within a multidimensional sustainability framework, mechanical and thermal performance should not be interpreted solely based on pore-structure parameters or any individual BET measurement. Instead, pore volume, specific surface area, average pore diameter, fiber–matrix interactions, and hydration-product formation mechanisms should be evaluated collectively.

SEM and BET observations indicated that the incorporation of kapok fiber, PC and WTRP increased microstructural heterogeneity through additional pores and weakened interfacial transition zones, which explains the observed reduction in compressive strength. Nevertheless, the XRD and TG-DSC findings indicated that the binder system maintained the formation and thermal stability of the principal strength-contributing hydration products. Therefore, despite the development of a more porous and heterogeneous microstructure, a sufficiently continuous load-bearing matrix was preserved, as demonstrated by the 28-day compressive strengths ranging from 29.59 to 41.27 MPa. Meanwhile, the crack-bridging and stress-transfer mechanisms of kapok fiber enhanced flexural performance, whereas the hollow fiber structure, petroleum-coke-induced discontinuities in the mineral skeleton, and low-conductivity rubber regions restricted heat transfer. Accordingly, the developed composites should not be regarded as conventional thermal-insulation materials or classified as structural mortars solely on the basis of compressive strength. Instead, they may be considered high-strength, thermally improved masonry-mortar candidates, particularly for wall systems requiring a balance among load-transfer capacity, resistance to flexural cracking, and reduced heat transmission. Further evaluation of bond strength, water absorption, shrinkage, vapor permeability, durability, and full-scale masonry behavior is required to confirm this application potential.

**Figure 20:**
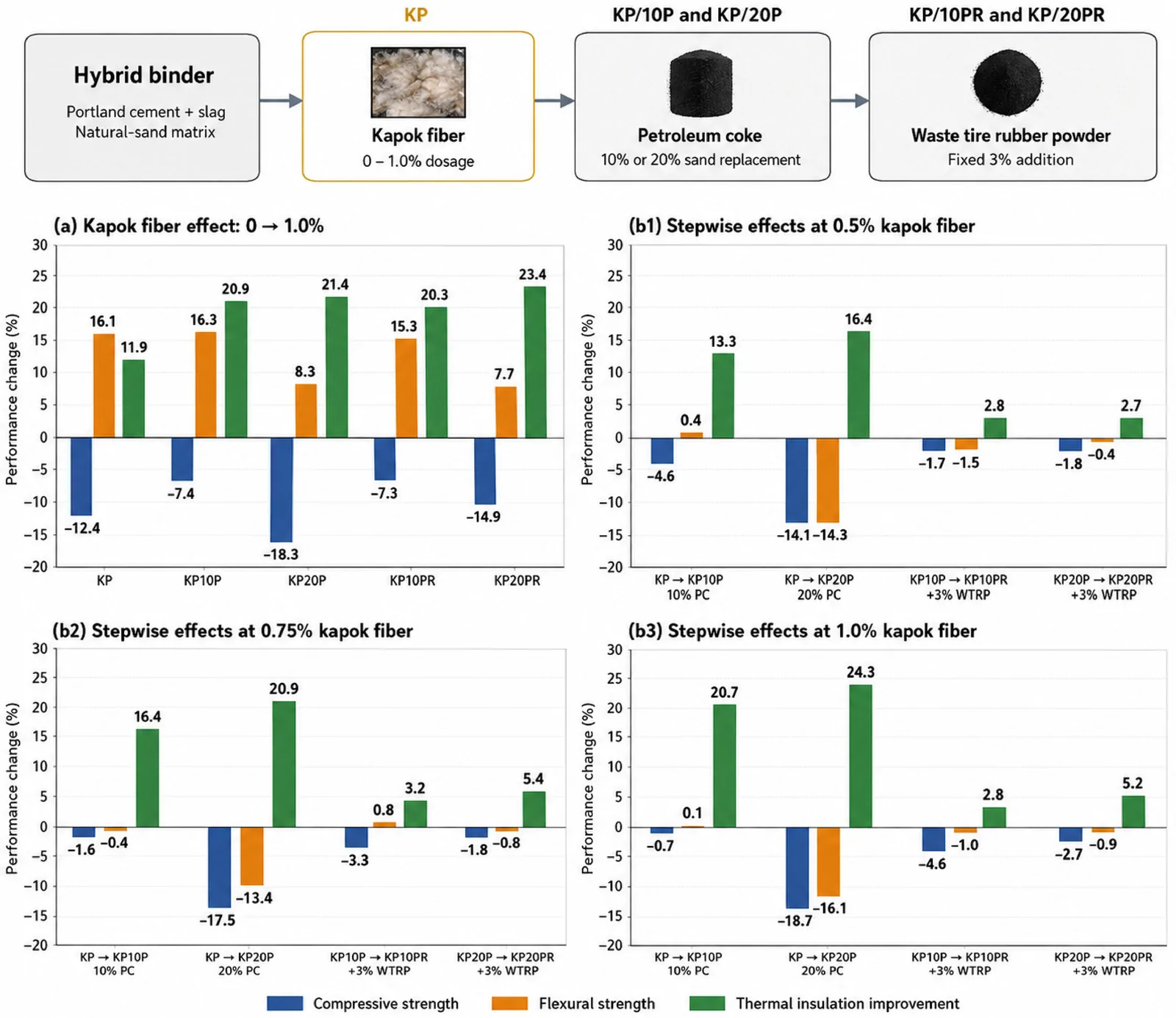
Quantitative assessment of the individual and stepwise combined contributions of kapok fiber, PC and WTRP to mechanical and thermal-insulation performance.

The incremental contribution analysis presented in Figure 20 quantitatively demonstrates the individual and combined effects of kapok fiber, PC and WTRP on the mechanical and thermal performance. Increasing the kapok fiber content from 0 to 1.0% reduced compressive strength by 7.4–16.3% across all mixture groups, while increasing flexural strength and thermal insulation performance by 7.7–16.3% and 11.0–23.4%, respectively. The 10% PC replacement improved thermal insulation by 13.1–20.7%, depending on the kapok fiber dosage, while producing changes of only −0.7% and +0.1% in compressive and flexural strengths, respectively, at 1.0% kapok fiber. In contrast, 20% PC replacement increased thermal insulation by 16.4–24.3% but caused more pronounced reductions in compressive and flexural strengths. The incorporation of WTRP into the PC-containing mixtures provided an additional 2.0–5.4% improvement in thermal insulation, generally accompanied by limited reductions in mechanical performance. These findings indicate that kapok fiber contributes primarily to flexural and thermal performance, whereas PC and WTRP provide complementary effects by restricting heat-transfer pathways. Nevertheless, particularly at higher PC contents, the combined system involves a distinct trade-off between mechanical and thermal performance. The results demonstrate that kapok fiber limited heat conduction through its hollow lumen structure while enhancing flexural performance through crack bridging and stress transfer. Petroleum coke disrupted the continuity of the dense mineral aggregate skeleton, creating more tortuous heat-transfer pathways, whereas waste tire rubber powder provided additional thermal insulation owing to its low thermal conductivity. However, the weakened particle–matrix interfaces associated with these waste-derived constituents reduced mechanical strength. Their combined incorporation generated a heterogeneous and tortuous heat-transfer network, while the crack-bridging action of kapok fiber partially compensated for the adverse effects of PC and WTRP on flexural performance.

## 4. Conclusions

In this study, the effects of kapok fiber, PC and WTRP on the mechanical, thermal conductivity, microstructural, phase composition, pore structure, and thermal behavior of the mortars were investigated. This synergistic system provides a new approach for the combined utilization of the bio-based and waste-derived contents, leading to enhanced insulation and flexural performance and offering valuable insights for improving environmental sustainability. The results obtained can be summarized as follows:

- Compared to the individual addition of kapok fiber, WTRP and PC, the synergistic effect of these bio-based and waste-derived contents significantly decreased the thermal conductivity of alkali-activated slag mortars. When kapok fiber, WTRP and PC were incorporated at 1.0%, 3.0% and 20% respectively, thermal insulation increased by 36.8%. Moreover, the reduction in flexural strength was negligible at 3.44%.
- Increasing the kapok fiber content consistently and without exception resulted in simultaneous enhancements in both flexural and thermal insulation performance across all sustainable mortar formulations explored. Similarly, the incorporation of PC and waste rubber powder enhanced the thermal insulation characteristics of the sustainable mortars while maintaining flexural performance with only limited strength losses. As anticipated, compressive strength decreased markedly.
- SEM investigations indicated that kapok fibers created a bridging effect inside the matrix; however, with the increasing incorporation of PC and WTRP, more heterogeneous matrix regions, voids around the fibers, and the formation of loose microstructures in particular areas were seen. These microstructural changes were found to be consistent with the mechanical strength results.
- XRD examination revealed that the main phases in all samples were quartz (Q), C-S-H gel phase, and portlandite (CH). The incorporation of PC and WTRP did not induce the formation of a new crystalline phase; instead, at high additive content levels, it caused a relative decrease in the C-S-H gel phase and an enhancement of the CH phase. This situation has demonstrated a correlation with the noted reductions in compressive strength.
- BET analyses indicated that the additives substantially modified pore characteristics. In mixtures containing 20% PC, the specific surface area increased by 55%, whereas the average pore diameter diminished by roughly 60%; the synergistic application of PC and WTRP markedly enhanced the total pore volume, thereby improving thermal insulation performance.
- The synergistic mechanism of the suggested mortar system was attributed to the complementary functions of the incorporated constituents. The hollow lumen structure of kapok fibers, along with the low thermal conductivity of PC and WTRP, created a more tortuous and heterogeneous heat-transfer pathway, thereby diminishing heat transfer within the mortar matrix. Simultaneously, the crack-bridging effect of kapok fibers mitigated the loss of flexural performance associated with increased porosity and weaker interfacial transition zones resulting from the waste-derived additives. These complementary mechanisms explain the observed synergistic relationship between pore structure, thermal insulation, and flexural performance.
- TG-DSC examinations indicated that all samples exhibited similar thermal degradation behavior. In mixtures comprising PC and WTRP, higher total mass losses were noted; however, it was concluded that the additives did not modify the fundamental reaction mechanism of the system. The TG-DSC results, consistent with SEM, XRD, and BET analysis, indicated that the additions affected thermal behavior by modifying the microstructure and pore structure.

This study demonstrated the synergistic mechanism of the bio-based and waste-derived contents in alkali-activated slags at the mortar scale. SEM, XRD, BET and TG-DSC analyses provided valuable insights into the synergistic behavior. While the results are encouraging, further optimization is still required. Although BET analysis enabled significant information on the nano-scale pore system, it could not directly quantify micron-scale interfacial voids and defects surrounding the fibers. Future work combining BET with mercury intrusion porosimetry (MIP), X-ray micro-computed tomography (micro-CT), or advanced microscopic techniques would enable a more comprehensive multi-scale characterization of the pore network and fiber–matrix interface. Long-term durability was also beyond the scope of this study and should be investigated in future research. The conclusions are limited to the designed mixture compositions after 28-day water curing, and broader experimental designs involving multiple curing regimes and durability condition variables are required to develop predictive models for the strength and thermal performance of kapok-fiber-reinforced PC- and WTRP-incorporated hybrid slag mortars.

## Figures and Tables

**Table 1:** Chemical composition and physical parameters of the utilized cement and slag.

	Cement	Slag
**Chemical composition (wt/wt %)**		
SiO_2_	18.7	34.71
Al_2_O_3_	4.8	9.9
Fe_2_O_3_	3.1	0.96
CaO	63	3.25
MgO	2.2	5.21
SO_3_	2.9	0.54
K_2_O	0.9	1.2
Na_2_O	0.2	0.22
Cl^−^	0.01	0.0083
**Physical properties**		
Setting time (min.) Initial	180	-
Final	240	-
Density (g/cm^3^)	3.11	2.91
Specific surface area (cm^2^/g)	3900	5258
Volume stability (mm)	1	-

**Table 2:** The characteristics of the utilized kapok fiber.

	Kapok Fiber
**Physical properties**	
Density (gr/cm^3^)	0.29
Moisture (%)	6–8
Length (mm)	20–35
Diameter (µm)	15–20
**Mechanical properties**	
Tensile strength (MPa)	1–2
Initial modules (GPa)	0.1–0.2
**Thermal insulation coefficient (W/m·K)**	**0.040–0.060**

**Table 3:** Mix proportions of designed mortar samples.

Mix	KP Fiber (gr)	Cement (gr)	Slag (gr)	Sand (gr)	Water (ml)	PC (gr)	WTRP (gr)	Lithium Carbonate (Li_2_CO_3_) (gr)
KP-0	-	225	225	1350	225	-	-	6.75
KP-0.5	2.25	225	225	1350	225	-	-	6.75
KP-0.75	3.375	225	225	1350	225	-	-	6.75
KP-1	4.5	225	225	1350	225	-	-	6.75
KP/10P-0	-	225	225	1215	225	135	-	6.75
KP/10P-0.5	2.25	225	225	1215	225	135	-	6.75
KP/10P-0.75	3.375	225	225	1215	225	135	-	6.75
KP/10P-1	4.5	225	225	1215	225	135	-	6.75
KP/20P-0	-	225	225	1080	225	270	-	6.75
KP/20P-0.5	2.25	225	225	1080	225	270	-	6.75
KP/20P-0.75	3.375	225	225	1080	225	270	-	6.75
KP/20P-1	4.5	225	225	1080	225	270	-	6.75
KP/10PR-0	-	225	225	1215	225	135	13.5	6.75
KP/10PR-0.5	2.25	225	225	1215	225	135	13.5	6.75
KP/10PR-0.75	3.375	225	225	1215	225	135	13.5	6.75
KP/10PR-1	4.5	225	225	1215	225	135	13.5	6.75
KP/20PR-0	-	225	225	1080	225	270	13.5	6.75
KP/20PR-0.5	2.25	225	225	1080	225	270	13.5	6.75
KP/20PR-0.75	3.375	225	225	1080	225	270	13.5	6.75
KP/20PR-1	4.5	225	225	1080	225	270	13.5	6.75

**Table 4:** The R^2^ of the linear correlation coefficient for all mixture groups.

		R^2^	F	Sig.
KP Group	CS-FS	0.921	23.347	0.040
	CS-TC	0.946	35.200	0.027
	FS-TC	0.976	82.773	0.012
KP-10P Group	CS-FS	0.938	30.339	0.031
	CS-TC	0.933	27.711	0.034
	FS-TC	0.975	76.32	0.013
KP-20P Group	CS-FS	0.878	14.405	0.063
	CS-TC	0.95	37.762	0.025
	FS-TC	0.971	68.085	0.014
KP-10PR Group	CS-FS	0.915	21.458	0.044
	CS-TC	0.948	36.684	0.026
	FS-TC	0.982	109.456	0.009
KP-20PR Group	CS-FS	0.904	18.767	0.049
	CS-TC	0.972	69.505	0.014
	FS-TC	0.977	84.483	0.012

## Data Availability

The original contributions presented in this study are included in the article. Further inquiries can be directed to the corresponding author.

## Abbreviations

The following abbreviations are used in this manuscript:

PC	Petroleum coke
KP	Kapok
WTRP	Waste tire rubber powder
Li_2_CO_3_	Lithium carbonate
EU	The European Union
CH	Portlandite
C-S-H	Calcium silicate hydrate
C-(A)-S-H	Aluminum-containing calcium silicate hydrate
ITZ	The fiber–matrix interface
Q	Quartz
SEM	Scanning electron microscope
BET	Surface area
XRD	X-ray diffraction
TG-DSC	Thermogravimetry and differential scanning calorimetry
CS	Compressive strength
FS	Flexural strength
TC	Thermal conductivity

## References

[1] Gagg C.R. (2014). Cement and concrete as an engineering material: An historic appraisal and case study analysis. Eng. Fail. Anal..

[2] Tiwari A., Singh S., Nagar R. (2016). Feasibility assessment for partial replacement of fine aggregate to attain cleaner production perspective in concrete: A review. J. Clean. Prod..

[3] Alsaif A.S., Alqarni A.S. (2024). Characteristics of concrete incorporating construction and demolition waste aggregate and post-consumer tires rubber and steel fibers. Constr. Build. Mater..

[4] Bu C., Tan B., Wu Q., Qiao Y., Sun Y., Yu L., Yang Q. (2023). Activation method and reuse of waste concrete powder: A review. Sustainability.

[5] Zamanian M., Salimi M., Payan M., Noorzad A., Hassanvandian M. (2023). Development of high-strength rammed earth walls with alkali-activated ground granulated blast furnace slag (GGBFS) and waste tire textile fiber (WTTF) as a step towards low-carbon building materials. Constr. Build. Mater..

[6] Wang X., Li F., Gao F., Wang Y., Li M., Wang J., Guo X., Nikolayevich K.S., Nikolaevich L.S. (2025). Characteristics of alkali-activated slag mortar under combined freeze-thaw cycles and sulfate exposure: Microstructural and mechanical insights. Constr. Build. Mater..

[7] Yang Z., Shi P., Zhang Y., Li Z. (2022). Influence of liquid-binder ratio on the performance of alkali-activated slag mortar with superabsorbent polymer. J. Build. Eng..

[8] Susurluk G., Sarıkaya H., Bostanci L. (2026). Effect of Bio-based Natural Kapok Fiber on Properties of Sustainable Mortars Containing Slag and Fly Ash as a Partial Replacement of Portland Cement. Adv. Civ. Eng..

[9] Perdana S., Vielle M. (2025). Industrial European regions at risk within the Fit for 55: How far implementing CBAM can mitigate?. Renew. Sustain. Energy Transit..

[10] Wei T., Kallbekken S. (2024). Carbon leakage from aviation under the European Union Fit for 55 policies. Transp. Res. Part D Transp. Environ..

[11] Provis J.L., Palomo A., Shi C. (2015). Advances in understanding alkali-activated materials. Cem. Concr. Res..

[12] Örklemez E., İlkentapar S., Durak U., Gülçimen S., Uzal N., Uzal B., Karahan O., Atiş C.D. (2024). Characterizing boron-enhanced one-part alkaline-activated mortars: Mechanical properties, microstructure and environmental impacts. Constr. Build. Mater..

[13] Polydorou T., Constantinides G., Neocleous K., Kyriakides N., Koutsokeras L., Chrysostomou C., Hadjimitsis D. (2020). Effects of pre-treatment using waste quarry dust on the adherence of recycled tyre rubber particles to cementitious paste in rubberised concrete. Constr. Build. Mater..

[14] Zhang R., Wang H., Ji J., Suo Z., Ou Z. (2022). Influences of different modification methods on surface activation of waste tire rubber powder applied in cement-based materials. Constr. Build. Mater..

[15] Zhu Z., Lu Y., Zhou M. (2023). Functionalized waste tire rubber powders for mitigating leaching of heavy metal ions and enhancing photocatalytic properties of rubberized cement mortar. J. Build. Eng..

[16] Zhang P., Wang X., Wang J., Zhang T. (2022). Workability and durability of concrete incorporating waste tire rubber: A review. J. Renew. Mater..

[17] Nemenova V., Abedini A., Liliedahl T., Engvall K. (2014). Co-gasification of petroleum coke and biomass. Fuel.

[18] Lv J., Wei X., Zhang Y., Zong Z. (2018). Mild oxidation of Yanshan petroleum coke with aqueous sodium hypochlorite. Fuel.

[19] Bostancı L. (2020). A comparative study of petroleum coke and silica aerogel inclusion on mechanical, pore structure, thermal conductivity and microstructure properties of hybrid mortars. J. Build. Eng..

[20] Wang L., Quan H., Li Q. (2019). Effect of solid waste-petroleum coke residue on the hydration reaction and property of concrete. Materials.

[21] Chemanalyst (2023). Petroleum Coke Market Analysis: Global Industry Trends and Forecast 2015–2030.

[22] Tripathi N., Singh R.S., Hills C.D. (2019). Microbial removal of sulphur from petroleum coke (petcoke). Fuel.

[23] Yang Z.L., Yan J.J., Wang F.M. (2018). Pore structure of kapok fiber. Cellulose.

[24] Sun Q., Xu J., Lu C., Zhu S., Lin G., Fan M., Li J., Chen K. (2023). Green and sustainable kapok fiber as novel core materials for vacuum insulation panels. Appl. Energy.

[25] Susurluk G., Sarikaya H., Bostancı L. (2024). Utilization of natural kapok and coconut fiber in thermally insulated sustainable concrete design. Environ. Sci. Pollut. Res..

[26] Dong T., Jiang W., Liu Y., Wu Y., Qi Y., Li J., Ma Y., Ben H., Han G. (2020). A phase change material embedded composite consisting of kapok and hollow PET fibers for dynamic thermal comfort regulation. Ind. Crops Prod..

[27] Sheng G., Zhai J., Li Q., Li F. (2007). Utilization of fly ash coming from a CFBC boiler co-firing coal and petroleum coke in Portland cement. Fuel.

[28] Frías M., Jiménez-Mateos J.M., Pfretzschner J., Olmeda J., Rodríguez R.M., Sánchez de Rojas M.I. (2011). Development of blended cement mortars with acoustic properties using petroleum coke. Constr. Build. Mater..

[29] Pei S.L., Pan S.Y., Gao X., Fang Y.K., Chiang P.C. (2018). Efficacy of carbonated petroleum coke fly ash as supplementary cementitious materials in cement mortars. J. Clean. Prod..

[30] Fadiel A., Al Rifaie F., Abu-Lebdeh T., Fini E. (2014). Use of crumb rubber to improve thermal efficiency of cement-based materials. Am. J. Eng. Appl. Sci..

[31] Liu K., Zhao X., Kou Y., Yu Y., Xie T. (2023). Compressive stress-strain relationship and its variability of self-compacting concrete incorporating recycled aggregate. J. Mater. Civ. Eng..

[32] Hancharoen K., Kamhangrittirong P., Suwanna P. (2024). Improvement of natural fiber cement composite for roofing applications through addition of waste tire rubber: An investigation of the physical, mechanical, thermal, and acoustic properties. Clean. Mater..

[33] Eldin A.N., Senouci M.A. (1993). Rubber-tire particles as concrete aggregate. J. Mater. Civ. Eng..

[34] Topçu I. (1995). The properties of rubberized concretes. Cem. Concr. Res..

[35] Segre N., Joekes J. (2000). Use of tire rubber particles as addition to cement paste. Cem. Concr. Res..

[36] Benazzouk A., Douzane O., Mezreb K., Queneudec J. (2007). Thermal conductivity of cement composites containing rubber waste particles: Experimental study and modelling. Constr. Build. Mater..

[37] Bostancı L., Üstündağ Ö., Çelik Sola Ö., Uysal M. (2020). Effect of curing methods and scrap tyre addition on properties of mortars. J. Civ. Eng..

[38] Han Z., Li C., Lu Y., Li Z., Pan D., Cao X. (2026). Radiative thermal management materials and devices: Principles, advances and potential applications. Mater. Today.

[39] Lei J., Zheng S., Liu S., Pan D., Shin S., Liu C., Cao X., Shen C. (2026). Flexible SiO_2_/Co@CNT composite fiber membrane via in-situ growth: Monolithic construction for efficient thermal insulation and EMI shielding. Chem. Eng. J..

[40] Lertwattanaruk P., Suntijitto A. (2015). Properties of natural fiber cement materials containing coconut coir and oil palm fibers for residential building applications. Constr. Build. Mater..

[41] Hancharoen K., Kamhangrittirong P., Suwanna P. (2020). Enhancement of thermal and sound insulation properties of cement composite roofing tile by addition of nanocellulose coated pineapple fiber and modified rubber tire waste. Key Eng. Mater..

[42] Wongsa A., Kunthawatwong R., Naenudon S., Sata V., Chindaprasirt P. (2020). Natural fiber reinforced high calcium fly ash geopolymer mortar. Constr. Build. Mater..

[43] Poletanovic B., Janotka I., Janek M., Bacuvcik M., Merta I. (2021). Influence of the NaOH-treated hemp fibers on the properties of fly-ash-based alkali-activated mortars prior and after wet/dry cycles. Constr. Build. Mater..

[44] Evangelia T., Ioanna I.V., Stefanidou M. (2024). Effect of hemp fibers and crystalline admixtures on the properties and self-healing efficiency of lime and clay-based mortars. J. Build. Eng..

[45] De Silva G.H.M.J., Naveen P. (2024). Effect of rice husk ash and coconut coir fiber on cement mortar: Enhancing sustainability and efficiency in buildings. Constr. Build. Mater..

[46] (2009). Methods of Testing Cement—Part 1: Determination of Strength.

[47] Kaya M., Atabey İ.İ., Çelikten S. (2026). Physical and microstructural characterization of wood ash-based geopolymer mortars with ceramic powder under different curing regimes. Constr. Build. Mater..

[48] Tsai C.J., Wu B.W., Hua C.Y., Wu Y.H., Lin W.T. (2026). Influence of alkali equivalent and aggregate type on the volumetric behavior of alkali-activated slag mortar during plastic and hardened phases. Mater. Today Adv..

[49] Bostanci L., Celik Sola O. (2018). Mechanical properties and thermal conductivity of aerogel-incorporated alkali-activated slag mortars. Adv. Civ. Eng..

[50] Bostanci L. (2020). Synergistic effect of a small amount of silica aerogel powder and scrap rubber addition on properties of alkali-activated slag mortars. Constr. Build. Mater..

[51] Sarikaya H., Susurluk G., Bostancı L. (2026). The role of kapok fiber in modifying the strength and thermal properties of cement and silica fume mortars: An experimental approach. Teh. Vjesn..

[52] Gao J., Wang Z., Zhang T., Zhou L. (2017). Dispersion of carbon fibers in cement-based composites with different mixing methods. Constr. Build. Mater..

[53] (2000). Methods of Test for Mortar for Masonry-Part 11: Determination of Flexural and Compressive Strength of Hardened Mortar.

[54] Juarez-Alvarado C.A., Magniont C., Escadeillas G., Terán-Torres B.T., Rosas-Diaz F., Valdez-Tamez P.L. (2022). Sustainable proposal for plant-based cementitious composites: Evaluation of their mechanical, durability, and comfort properties. Sustainability.

[55] Ziada M., Erdem S., Gonzales-Lezcano R.A., Tammam Y., Unkar I. (2023). Influence of various fibers on the physico-mechanical properties of a sustainable geopolymer mortar based on metakaolin and slag. Eng. Sci. Technol. Int. J..

[56] Thapa K., Sedai S., Paudel J., Gyawali T.R. (2024). Investigation on the potential of Eulaliopsis binata (babiyo) as a sustainable fiber reinforcement for mortar and concrete. Case Stud. Constr. Mater..

[57] Rashad A.M. (2016). A comprehensive overview about recycling rubber as fine aggregate replacement in traditional cementitious materials. Int. J. Sustain. Built Environ..

[58] Olmeda J., Sánchez de Rojas M.I., Frías M., Donatello S., Cheeseman C.R. (2013). Effect of petroleum (pet) coke addition on the density and thermal conductivity of cement pastes and mortars. Fuel.

[59] Olanrewaju O., Oladele I.O., Adelani S.O. (2025). Natural fiber biocomposites: A comprehensive review of current developments and material properties of plant and animal fiber reinforcements in cement concrete, and ultra-high-performance concrete. Next Mater..

[60] Aygörmez Y. (2022). Performance of different binders doped Portland cement-based mortars using volcanic slag, petroleum coke and EPS foam aggregates. Constr. Build. Mater..

[61] Ramakrishna G., Sundararajan T. (2005). Studies on the durability of natural fibers and the effect of corroded fibers on the strength of mortar. Cem. Concr. Compos..

[62] García G., Cabrera R., Rolón J., Pichardo R., Thomas C. (2024). Natural fibers as reinforcement of mortar and concrete: A systematic review from Central and South American regions. J. Build. Eng..

[63] Han Y., Shi M., Lee S., Lin R., Wang K.J., Wang X.Y. (2024). Application of porous luffa fiber as a natural internal curing material in high-strength mortar. Constr. Build. Mater..

[64] Çelik Sola Ö., Özyazgan C. (2017). Mechanical properties of mortar containing recycled asphalt. Građevinar.

[65] de Oliveira L.B., Marvila M.T., Vieira C.M.F., de Azevedo A.R.G. (2026). Fresh and hardened performance of geopolymer mortars reinforced with chemically treated açaí fibers. J. Build. Eng..

[66] Efendiyev G.M., Moldabayeva G.Z., Tuzelbayeva S.R., Buktukov N.S., Kozlovskiy A.L., Tileuberdi N., Kirisenko O.G. (2025). Enhancing cement mortar performance for construction and oil well applications using fiber reinforcement. ES Mater. Manuf..

[67] Tian S., Ma S., Chen H., Yao X., Xu X., Chen Z. (2025). The synergistic effect of mineral nanofibers on the microstructure evolution and macroscopic properties of one-part alkali-activated slag-fly ash mortar. J. Build. Eng..

[68] Tian S., Ma S., Chen H., Yao X., Chen J., Xu X. (2026). Performance and mechanism of diatomite-modified wood fiber reinforced mortar: Toward sustainable and durable building materials. J. Build. Eng..

[69] Benjeddou O., Aldahdooh M.A.A. (2026). Ground granulated blast furnace slag as a supplementary cementitious material in concrete: A comprehensive review. ES Mater. Manuf..

[70] Liu X., Wang D., Wang Z. (2026). Toughening mechanism of basalt fiber-reinforced SCMs-modified eco-friendly mortar: Flexural and fracture behavior. Constr. Build. Mater..

[71] Cheng C., Zhou J., Wang X., Ding L., Fan J., Hao X., Xie J., Wu Z., Liu D. (2026). Influence of multi-scale fibers on mechanical properties and shrinkage behavior in alkali-activated slag/fly ash geopolymer mortar. Constr. Build. Mater..

[72] Mo J., Lu H., Ren F., Lai M., Ho J.C.M., Guo S., Sun R., Tian S., Xiong J. (2026). Impact of waste rubber powder size and replacement ratio on the compressive strength anisotropy and pore structure of 3D printed concrete. J. Build. Eng..

[73] (2018). Building Earthquake Code.

